# Gata6^+^ resident peritoneal macrophages promote the growth of liver metastasis

**DOI:** 10.1038/s41467-022-32080-y

**Published:** 2022-07-29

**Authors:** Mokarram Hossain, Raymond Shim, Woo-Yong Lee, Arlene H. Sharpe, Paul Kubes

**Affiliations:** 1 Department of Physiology and Pharmacology, University of Calgary, 3330 Hospital Drive NW, Calgary, AB T2N 4N1 Canada; 2 Snyder Institute for Chronic Diseases, University of Calgary, 3330 Hospital Drive NW, Calgary, AB T2N 4N1 Canada; 3 Department of Immunology, Blavatnik Institute, Harvard Medical School, and Evergrande Centre for Immunological Diseases, Harvard Medical School and Brigham and Women’s Hospital, Boston, MA 02115 USA; 4 Broad Institute of MIT and Harvard, Cambridge, MA 02142 USA; 5 Department of Pathology, Brigham and Women’s Hospital, Boston, MA 02115 USA; 6 Department of Microbiology and Immunology, University of Calgary, 3330 Hospital Drive NW, Calgary, AB T2N 4N1 Canada; 7 Present Address: Early Oncology, AstraZeneca, Gaithersburg, MD 20878 USA

**Keywords:** Tumour immunology, Peritoneal macrophages, Imaging the immune system

## Abstract

Emerging evidence suggests that resident macrophages within tissues are enablers of tumor growth. However, a second population of resident macrophages surrounds all visceral organs within the cavities and nothing is known about these GATA6^+^ large peritoneal macrophages (GLPMs) despite their ability to invade injured visceral organs by sensing danger signals. Here, we show that GLPMs invade growing metastases that breach the visceral mesothelium of the liver via the “find me signal”, ATP. Depleting GLPMs either by pharmacological or genetic tools, reduces metastases growth. Apoptotic bodies from tumor cells induces programmed cell death ligand 1 (PD-L1) upregulation on GLPMs which block CD8^+^ T cell function. Direct targeting of GLPMs by intraperitoneal but not intravenous administration of anti-PD-L1 reduces tumor growth. Thermal ablation of liver metastases recruits huge numbers of GLPMs and enables rapid regrowth of tumors. GLPMs contribute to metastatic growth and tumor recurrence.

## Abstract (web summary)

GLPMs represent a population of fetal liver derived large peritoneal cavity Gata6+ macrophages, with the capacity to invade the tissues they surround. Here, in experimental colon cancer liver metastasis models, the authors show that GLPMs invade liver metastasis directly from the peritoneum, promoting liver metastases growth.

## Introduction

A hallmark feature of the tumor microenvironment (TME) is the presence of many immune cells^1^. Despite the presence of these immune cells, an established tumor is usually not eradicated but rather aided in its growth, suggesting a net immunosuppressive environment^2^. One of the most abundant immune cells in tumors are the macrophages, commonly known as tumor-associated macrophages (TAMs)^2^. TAMs can promote tumor growth when skewed towards a repair macrophage phenotype. Indeed, in breast cancer, TAMs promote invasiveness of the primary tumor and can help tumor cells evade immunity^1^. In colorectal cancer, TAMs increase in number, suppress immune cells, and promote angiogenesis^3^. Fate-mapping approaches have revealed that highly motile inflammatory monocytes are the source of recruited tumor-associated macrophages in models of murine breast cancer^4–6^. By contrast, most tissue-resident macrophages are sessile^7^ and less likely to invade tumors. As such, the focus has been primarily on blocking monocyte and other myeloid cell recruitment from the vasculature while other macrophages and other non-vascular routes of entry have been given little consideration.

However, there is some recent evidence that tissue-resident macrophages may also enter the TME. Indeed, Müller and colleagues provided some evidence that microglia, the tissue-resident macrophages in the brain, could be a source of TAMs in brain tumors^8^. Moreover, Zhu et al. showed using an elegant fate-mapping technique, that pancreatic ductal adenocarcinoma contains both monocyte-derived and embryonically derived tissue resident macrophages^9^. Another recent report showed that embryonic-derived tissue-resident macrophages accumulate in colon adenoma^10^ but because these cells are sessile, it is unknown how they mobilize to the tumors. Interestingly, there is another source of resident macrophages found in body cavities, including the peritoneum^11^, that surround the visceral organs and associated tumors. This population of fetal liver-derived large peritoneal cavity macrophages (GLPMs) express Gata6 and can therefore be easily distinguished from all other macrophages and immune cells. In contrast to the sessile tissue-resident macrophages in most organs, imaging the millions of Gata6^+^ cavity macrophages that occupy these cavities unveiled that they are very motile, moving through the fluid phase of the peritoneum unattached, with the capacity to invade the tissues they surround^12^. These cells are not restricted to the peritoneum but can be found in other cavities including the pleura and the pericardium^13,14^. Indeed, recent reports clearly showed that cavity macrophages in peritoneum and pericardium invade injured organs directly from the cavity (not via vasculature) and contribute significantly to the repair process^14,15^. Whether these cavity macrophages with holistic repair potential can similarly invade growing cancers in visceral organs remains unclear. Lack of previous reports of GLPMs in the tumors of visceral organs is not surprising given the fact that these macrophages have been reported to downregulate their prototypical marker Gata6 after leaving their natural environment^13,14^, making it impossible to specifically track these cells in human and mouse models of cancer.

Immunotherapy that blocks the interaction between programmed cell death protein 1 (PD-1) and programmed cell death ligand 1 (PD-L1) has been very effective against different types of cancers such as melanoma, Hodgkin’s lymphoma, colorectal cancer and non-small-cell lung cancer^16^. Macrophages within tumors are well known to express both PD-1 and PD-L1^17,18^. When macrophages express PD-1, they can directly engage PD-L1 expressed on tumor cells thereby inhibiting macrophage-mediated tumor cell killing^17^. Moreover, PD-L1-expressing macrophages indirectly protect tumor cells by blocking cytotoxic activity of PD-1-expressing CD8^+^ T cells^19^. Macrophages have been shown to express PD-L1 in response to multiple stimuli including tumor cell-derived CCL9^18^, hypoxic tumor microenvironment^20^ and uptake of nucleic acid rich tumor exosomes^21^. Most of these findings are restricted to subcutaneous implantation models of tumor^22^. Data on the role of TAMs in metastatic tumor growth in the liver is very limited and checkpoint inhibitors as therapy for liver metastases has been less studied and appears to be less effective^23^.

In this study, we show a source and subtype of fully mature macrophages that invade liver metastases directly from the peritoneum by sensing tumor-induced mesothelial damage. These macrophages, characterized by their expression of the zinc finger transcription factor Gata6^24^, are commonly known as the large peritoneal macrophages^11^ but upon entering tissue/tumor they down regulate this molecule. GLPMs upregulate PD-L1 upon taking up tumor cell-derived apoptotic bodies within the TME and promote the growth of the liver metastases. Additionally, PD-L1-expressing GLPMs are poorly targeted by intravascularly administered PD-L1 blocking antibody but can be improved by intraperitoneal administration. Thermal ablation, a common clinical practice to treat unresectable liver metastases, causes massive infiltration of GLPMs that significantly increase the regrowth of the ablated tumors.

## Results

### CT26 liver metastases recruit GLPMs

**Fig. 1:**
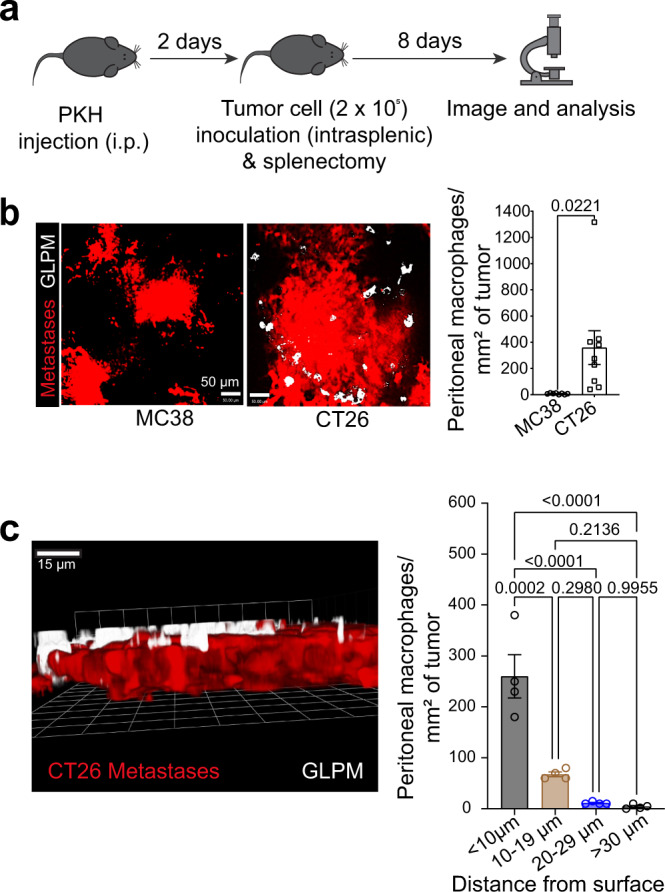
CT26 liver metastases recruit Gata6^+^ large peritoneal macrophages (GLPMs). **a** PKH dye injection and cancer metastasis model. **b** Representative confocal images (left; scale bar = 50 μm) of localization and quantification (right) of PKH^+^ GLPMs (White) in MC38 (Red) and CT26 (Red) liver metastases (*n* = 8 for MC38, *n* = 9 for CT26; from three independent experiments), *P* values were calculated using two-tailed unpaired *t* test *P* = 0.0221. **c** Representative 3D imaging of the distribution of GLPMs (white) and CT26 cancer metastasis (red; left) and quantification of GLPM penetration into metastasis (right) (*n* = 4; from one experiment), *P* values were calculated using ordinary one-way ANOVA with Tukey’s multiple comparisons test, *P* = 0.0002 < 10 µm vs 10–19 µm, *P* < 0.0001 < 10 µm vs 20–29 µm, *P* < 0.0001 < 10 µm vs >30 µm, *P* = 0.2980 10–19 µm vs 20–29 µm, *P* = 0.2136 10–10 µm vs 30 µm, *P* = 0.9955 20–29 µm vs >30 µm. All graphs are presented as mean ± SEM. Source data are provided with this paper.

Colorectal cancer frequently metastasizes to the liver^25^. We used the most commonly used and reproducible model of experimental liver metastasis^26,27^ to study the role of peritoneal macrophages in the tumor microenvironment. The spleen is surgically exposed for direct injection of tumor cells. We wait 1 min to allow for tumor cells to enter the bloodstream and circulate to the liver. This is followed by splenectomy to avoid tumor growth in the spleen. We tested two different colorectal cancer cell lines (CT26 and MC38) and, in some experiments, also a melanoma cell line (B16F10 melanoma cells) and a breast cancer cell line (4T1 breast cancer cells) in this syngeneic mouse model of liver metastasis for potential GLPM recruitment. To track GLPMs for prolonged behaviors, we used a specialized formulation of PKH26 dye that when injected intraperitoneally is predominantly taken up by GLPMs (96%) and not macrophages in the blood, spleen, and bone marrow under homeostatic conditions (Supplementary Fig. 1a–c). The PKH is stored in vesicles inside GLPMs for weeks without affecting the behavior of these cells or inducing any untoward inflammation^28,29^. Nevertheless, we verified these results with a second GATA6 reporter system (described below). Two days post GLPM labeling, we performed our tumor metastasis model and liver metastases were evaluated 8 days later (Fig. 1a). CT26 liver metastases recruited many PKH^+^ GLPMs while MC38 liver metastases failed to recruit these same macrophages (Fig. 1b). Furthermore, we used 3D imaging to quantify the depth of GLPM penetration within the sites of metastases. The majority of GLPMs localize to the surface of the metastases and do not penetrate more than 20 µm (Fig. 1c; Supplementary movie 1). Flow cytometry revealed that the PKH^+^ macrophages in the tumor environment were GLPMs although the level of Gata6 was downregulated (Supplementary Fig. 1d, e). We further confirmed our findings by generating Gata6^H2B-Venus^ bone marrow chimeric mice that express the Venus reporter only in the cavity macrophages (Supplementary Fig. 1f). Our previous study demonstrated that cavity macrophages downregulate the expression of Gata6 as they move into a different tissue environment and thus lose their reporter signal^14^, confirmed using our reporter mouse. Therefore, we continued to identify GLPMs using our PKH26 dye labeling approach for subsequent experiments. Overall, these data demonstrate that GLPMs are recruited to tumor sites in CT26 but not MC38 liver metastases and subsequently downregulate their GATA6 transcription factor.

### Disruption of mesothelium is required for GLPM recruitment to liver metastases

**Fig. 2:**
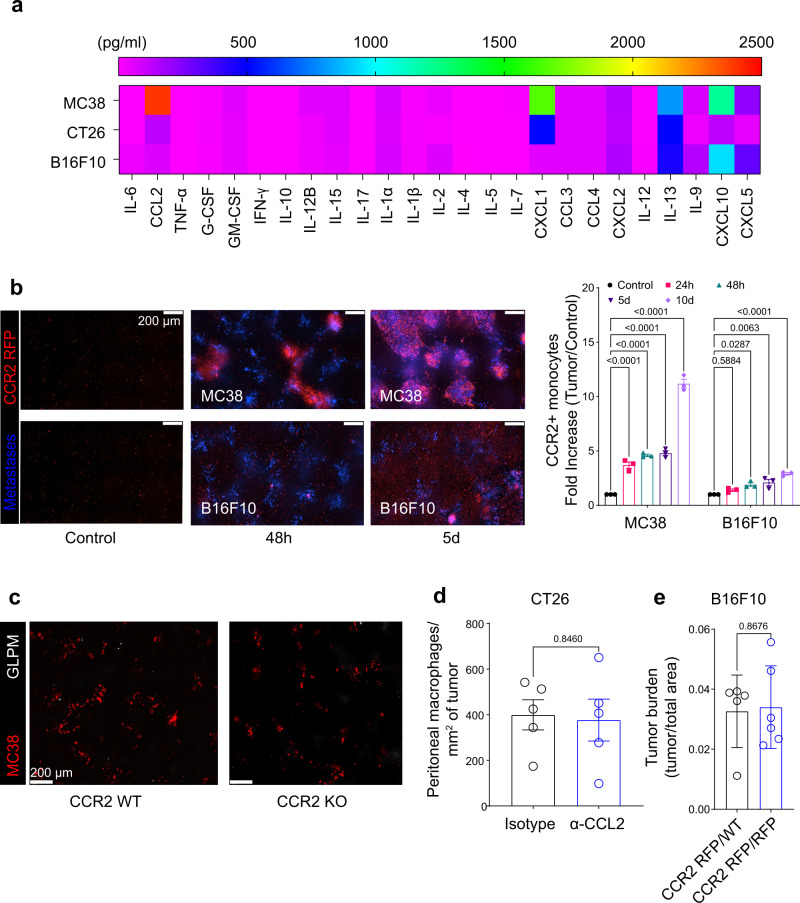
Monocytes do not contribute to GLPM recruitment to metastasis. **a** A heat map showing differential secretion of chemokine, cytokine and growth factors by MC38, CT26 and B16F10 cells as measured by a Luminex assay. **b** Representative intravital imaging (left; scale bar = 200 μm) and quantification (right) of CCR2 + monocyte (red) recruitment at 24 h, 48 h, 5 d and 10 d following MC38 or B16F10 liver metastasis (blue) (*n* = 3 mice per group), P values were calculated using ordinary two-way ANOVA with Tukey’s multiple comparisons test with individual variances computed for each comparison, *P* < 0.0001 MC38 control vs 24 h, 48 h, 5 d or 10 d. *P* = 0.5796 B16F10 control vs 24 h, *P* = 0.0217 B16F10 control vs 48 h, *P* = 0.0039 B16F10 control vs 5 d, *P* < 0.0001 B16F10 control vs 10 d. **c** Intravital microscopy showing LPM (white) and MC38 liver metastases (red) in WT vs CCR2 KO mice following MC38 liver metastasis (scale bar = 200 μm). **d** Quantification of peritoneal macrophage accumulation on CT26 cancer metastasis (*n* = 5 mice per group; from two independent experiments), *P* values were calculated using two-tailed unpaired *t* test, *P* = 0.8460. **e** Quantification of B16F10 tumor burden between CCR2 RFP/WT and CCR2 RFP/RFP mice (*n* = 5 for CCR2 RFP/WT, *n* = 6 CCR2 RFP/RFP; from two independent experiments), *P* values were calculated using two-tailed unpaired *t* test, *P* = 0.8676. All graphs are presented as mean ± SEM. Source data are provided with this paper.

To investigate why only CT26 but not MC38 metastases recruit GLPMs, we analyzed the chemokines, cytokines and growth factors that are secreted by these two cell lines. We could not identify any CT26-specific chemokines, cytokines or growth factors that could be attributed to the selective GLPM recruitment by CT26 metastases (Fig. 2a). In fact, MC38 tumors secreted more myeloid cell-specific chemokines, particularly CCL2 than CT26 cells (Fig. 2a) which led to monocyte recruitment in this tumor (Fig. 2b). To determine whether the monocyte recruitment somehow prevented or filled the niche otherwise occupied by GLMPs, we tested if MC38 metastases would recruit GLPMs in CCR2 knockout mice. No increase in GLMPs were seen in MC38 metastases in CCR2 KO mice (Fig. 2c). In CT26 metastasis, blocking monocyte recruitment with α-CCL2 antibody treatment did not alter GLPM levels on the tumor (Fig. 2d). Furthermore, tumor burden was not changed in our B16F10 metastasis model (Fig. 2e) or in MC38 metastasis model (Supplementary Fig. 2a) in the absence of monocyte recruitment (CCR2 KO) suggesting that monocytes had little contribution to tumor growth. These data suggest that the accumulation of GLMPs to the site of metastasis was independent of monocytes.

**Fig. 3:**
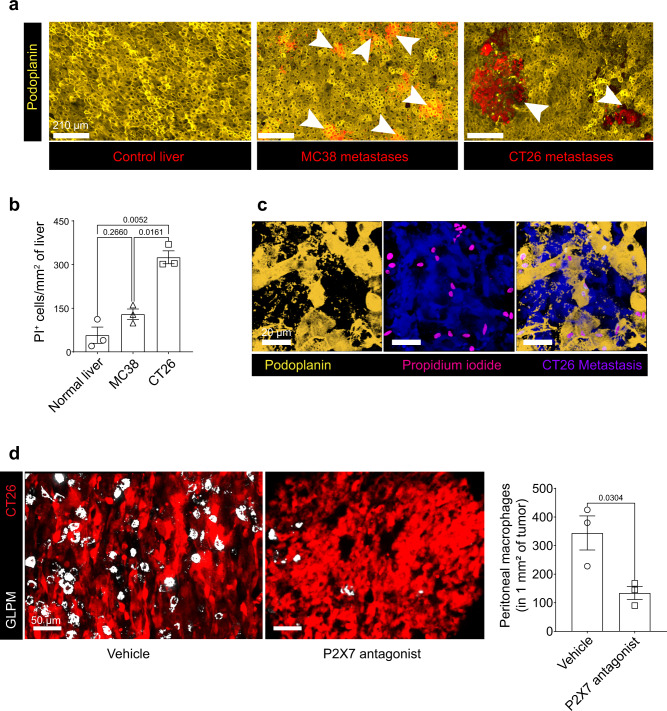
Mesothelium damage is required for GLPM recruitment to liver metastases. **a** Representative intravital images of the liver mesothelium (yellow) in control, MC38 or CT26 metastases (red; white arrows) (scale bar = 210 μm) (*n* = 5 per group; from two independent experiments). **b** Quantification of PI^+^ cells from normal, MC38 or CT26 liver (*n* = 3 mice per group; from one experiment), *P* values were calculated using ordinary two-way ANOVA with Tukey’s multiple comparisons test, *P* = 0.2660 normal liver vs MC38, *P* = 0.0052 normal liver vs CT26, *P* = 0.0161 MC38 vs CT26. **c** Representative image of colocalization of podoplanin^+^ (yellow) and PI^+^ (magenta) cells following CT26 liver metastases (blue) (scale bar = 20 µm) (*n* = 6; two independent experiments). **d** Representative intravital images (left; scale bar = 50 μm) and quantification (right) of GLPMs (white) within CT26 liver metastases (red) in mice treated with P2X7 receptor antagonist or vehicle (PBS) (*n* = 3 mice per group; one experiment), *P* values were calculated using two-tailed unpaired *t* test, *P* = 0.0304. All graphs are presented as mean ± SEM. Source data are provided with this paper.

The visceral mesothelium separates the peritoneal contents from visceral organs forming a physical barrier^30^. As such, we examined the mesothelial monolayer of the liver in both CT26 and MC38 metastases. CT26 metastases clearly disrupted the mesothelium revealing tumors (red) while the MC38 metastases grew within the liver but failed to breach the mesothelium and remained covered by the intact yellow mesothelium (Fig. 3a). When the liver mesothelium was disrupted mechanically by gentle physical scraping, GLPMs invaded the MC38 metastases (Supplementary Fig. 2b, c) suggesting a mesothelial breach is crucial for GLPM recruitment. Using imaging, we identified many propidium iodide positive (PI^+^) cells in the CT26 but not M38 metastases or in healthy livers (Fig. 3b, c; Supplementary Fig. 2d). Further analysis revealed that the vast majority of the PI^+^ cells were mesothelial cells as PI staining colocalized with podoplanin^+^ cells (Fig. 3c; Supplementary movie 2). GLPMs have been shown to invade the injured liver via the “find me signal”, ATP, released by dying cells^15^. Indeed, blockade of P2X7 receptor which binds ATP, significantly blocked the invasion of GLPMs into the liver metastases (Fig. 3d). These data show that danger signal release from mesothelial disruption is required for GLPM recruitment to tumor metastases.

### GLPMs promote the growth of CT26 liver metastases

**Fig. 4:**
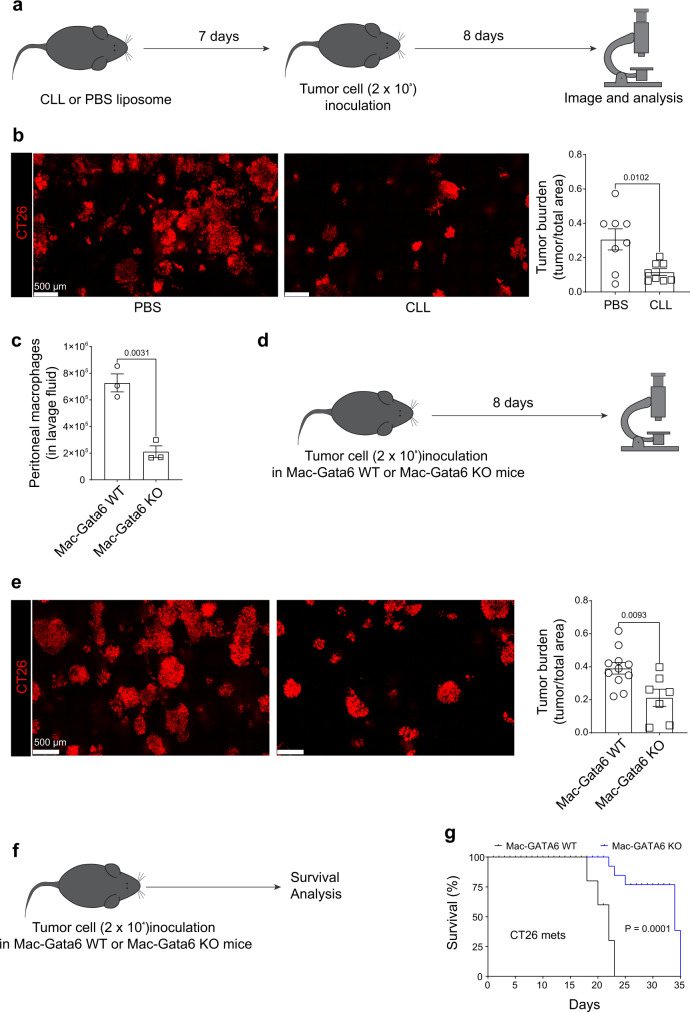
GLPMs promote the growth of CT26 liver metastases. **a** Treatment regime of low-dose (50 µl) of Clodronate-loaded liposome (CLL) or PBS loaded liposome prior to tumor cell inoculation. **b** Representative intravital images (left; scale bar = 500 μm) and quantification (right) of CT26 metastases (red) bearing liver of PBS or CLL liposome-treated mice with quantification (*n* = 8; from two independent experiments), *P* values were calculated using two-tailed unpaired *t* test, *P* = 0.0102. **c** Peritoneal macrophage number from Mac-Gata6 WT and Mac-Gata6 KO mice (*n* = 3; from one experiment), *P* values were calculated using two-tailed unpaired *t* test *P* = 0.0031. **d** Treatment regime of tumor cell inoculation in Mac-Gata6 WT or Mac-Gata6 KO mice. **e** Representative stitched images (left; scale bar = 500 μm) of CT26 metastases (red) bearing liver from Mac-Gata6 WT and Mac-Gata6 KO mice with quantification (right; *n* = 7 for Mac-Gata6 KO and *n* = 11 for Mac-Gata6 WT; from three independent experiments), *P* values were calculated using two-tailed unpaired *t* test, *P* = 0.0093. **f** Treatment regime of lower dose of tumor cell inoculation in Mac-Gata6 WT or Mac-Gata6 KO mice for survival analyses. **g** Survival curve of CT26 liver metastases bearing Mac-Gata6 WT and Mac-Gata6 KO mice (*n* = 10), *P* values were calculated using a Log-rank (Mantel–Cox) test, *P* = 0.001. All graphs are presented as mean ± SEM. Source data are provided with this paper.

To investigate the contribution of GLPMs to liver metastases, tumor growth was studied in peritoneal macrophage depleted mice as well as in macrophage-specific Gata6 knockout mice (Mac-Gata6 KO). GLPM depletion was performed using clodronate-loaded liposomes (CLL) at 7 days prior to initiating our cancer metastasis model (Fig. 4a). This 7-day delay was necessary because chlodronate administration induces initial inflammation that completely returned to baseline within 5 days. However, a single, low dose (50 µl) i.p. injection of CLL was 100% efficient at depleting all the resident peritoneal macrophages for up to three weeks (Supplementary Fig. 3a), whereas monocytes and other cells rapidly replenished the peritoneum. Importantly, at 7 days following injection with this dosage of CLL, liver CX3CR1^+^ subcapsular macrophage number (Supplementary Fig. 3b) and Kupffer cell number and function (Supplementary Fig. 3c) were not altered. GLPM depletion reduced the growth of CT26 liver metastases significantly, suggesting a pro-tumorigenic role for peritoneal macrophages (Fig. 4b). Nevertheless, we reconfirmed our findings with a second genetic approach to depleting GLPMs using a macrophage-specific Gata6 KO mouse (Mac-Gata6 KO). These mice have approximately 70% reduction of large peritoneal macrophages compared to their wild-type counterpart (Fig. 4c). The remaining 30% of large peritoneal macrophages lack any of the healing properties of GLPM^15^ and fail to replicate in response to various stimuli^31^. As cavity macrophages are the only macrophage population that express Gata6^14,15,24^ (Supplementary Fig. 3d), the Mac-Gata6 KO mouse strain is more specific but less efficient for GLPM depletion. Indeed, in the Mac-Gata6 KO mice (Fig. 4d) we observed that CT26 metastases grew slower compared to wild-type mice further confirming that recruited GLPMs were pro-tumorigenic (Fig. 4e) however the growth reduction was not as slow as when CLL liposome treatment was used.

Next, we developed a modified protocol of inoculating only 20,000 tumor cells that results in only a few liver metastases (similar to clinical cases) for survival analyses (Fig. 4f). We compared the survival following liver metastasis between Mac-Gata6 WT and Mac-Gata6 KO mice using this modified protocol (Fig. 4f). Mac-Gata6 KO mice survived significantly longer than their wild-type littermates when CT26 liver metastases was induced (Fig. 4g). Overall, these data suggest that GLPMs promote tumor growth following liver metastasis.

### GLPMs promote tumor growth by upregulating PD-L1 and suppressing CD8+ cells

**Fig. 5:**
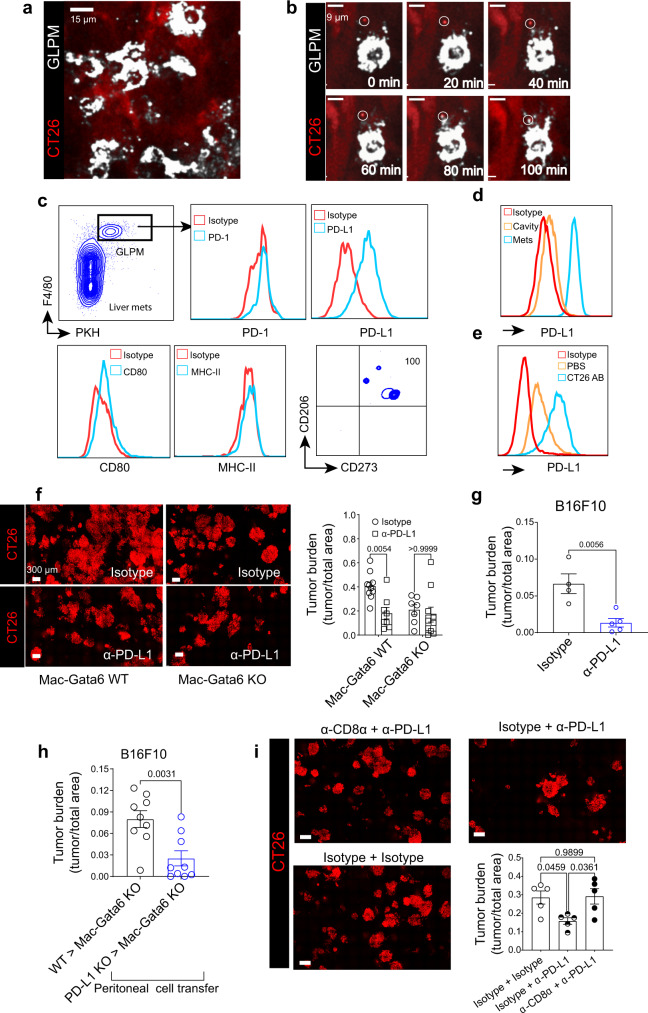
Expression of PD-L1 on GLPMs promote tumor growth by suppressing CD8+ T cells. **a** Association of GLPMs (white) to CT26 tumor cells (red) after liver metastasis (scale bar = 15 µm; *n* = 9; from three independent experiments). **b** Intravital imaging time-lapse of GLPM (green) uptake of CT26 tumor cell particle (red and circled) over 100 min (scale bar = 9 µm; *n* = 4; from two independent experiments). **c** Expression analyses of PD-1, PD-L1, CD80, MHC-II, CD206 and CD273 in GLPMs within the tumor microenvironment (*n* = 4 per group; from one independent experiment). **d** Expression of PD-L1 on tumor-associated GLPMs versus free-flowing cavity GLPMs (*n* = 3; from one experiment). **e** In vitro expression of PD-L1 on GLPMs following overnight incubation with CT26 apoptotic bodies (AB) (*n* = 6; from two independent experiment). **f** Representative intravital images (left; scale bar = 300 μm) and quantification (right) of CT26 tumor burden in the liver of Mac-Gata6 WT and Mac-Gata6 KO mice intraperitoneally treated with anti-PD-L1 blocking antibody (*n* = 10 for isotype Mac-Gata6 WT, *n* = 8 for anti-PD-L1 Mac-Gata6 WT, *n* = 8 for isotype Mac-Gata6 KO, *n* = 10 for anti-PD-L1 Mac-Gata6 KO; from three independent experiments), *P* values were calculated using an Ordinary two-way ANOVA with Bonferroni’s multiple comparisons test, with individual variances computed for each comparison, *P* = 0.054 Mac-Gata6 WT isotype vs anti-PD-L1, *P* > 0.9999 Mac-Gata6 KO isotype vs anti-PD-L1. **g** Tumor burden following B16F10 liver metastasis following treatment with isotype or anti-PD-L1 antibody (*n* = 4 for isotype, *n* = 5 for α-PD-L1; from two independent experiments), *P* values were calculated using two-tailed unpaired *t* test, *P* = 0.0056. **h** Tumor burden following peritoneal cell transfer of WT or PD-L1 KO GLPMs into Mac-Gata6 KO mice following B16F10 metastasis (*n* = 9 per group; from three independent experiments), *P* values were calculated using two-tailed unpaired *t* test, *P* = 0.0031. **i** Representative intravital images (left; scale bar = 300 µm) and quantification (right) of CT26 tumor burden (red) in the liver of wild-type mice treated with intraperitoneally administered anti-PD-L1 blocking antibody and/or CD8^+^ T cell depleting antibody (*n* = 5 per group; from two independent experiments), *P* values were calculated using an Ordinary one-way ANOVA with Tukey’s multiple comparisons test, *P* = 0.0459 isotype + isotype vs isotype + anti-PD-L1, *P* = 0.9899 isotype + isotype vs anti-CD8a+anti-PD-L1, *P* = 0.0361 isotype + anti-PD-L1 vs anti-CD8a vs anti-PD-L1. All graphs are presented as mean ± SEM. Source data are provided with this paper.

In the TME, we observed GLPMs adhered in close proximity to tumor cells (Fig. 5a) and, using intravital imaging, we could capture the interaction of GLPMs with iRFP-expressing tumor cell particles in real-time (Fig. 5b; Supplementary movie 3). These tumor particles were reminiscent of apoptotic bodies from the tumor cells as previously reported by Lai and colleagues^32^. Analysis of the expression of molecules that are known to be expressed by TAMs revealed that, in contrast to GLPM outside the tumor environment, GLPMs that were localized within the tumors upregulated checkpoint molecule PD-L1, but not PD-1, MHC-II or CD80 (Fig. 5c, d). Interestingly, all TME-associated GLPMs expressed CD206 and CD273 (Fig. 5c), indicating these macrophages were skewing towards an alternative macrophage phenotype^15^.

We further explored whether phagocytosis of apoptotic bodies is linked to PD-L1 upregulation in vitro. Co-incubation of freshly isolated resident peritoneal macrophages with CT26 cell-derived apoptotic bodies indeed upregulated PD-L1 in these macrophages. Simply harvesting Gata6^+^ cavity macrophages from the peritoneum and culturing them overnight in PBS was sufficient to upregulate PD-L1 (Fig. 5e), albeit less than those incubated with CT26 apoptotic bodies, suggesting that migration from the peritoneal environment induced PD-L1 expression which was further enhanced by apoptotic bodies. To test the functional relevance of these findings in vivo, we blocked PD-L1 in wild-type mice and noted a significant reduction in CT26 tumor burden (Fig. 5f). Anti-PD-L1 in Mac-Gata6 KO mice that already had less tumor burden, failed to further reduce tumor growth (Fig. 5f). Furthermore, anti-PD-L1 treatment could also significantly reduce tumor burden after B16F10 liver metastasis (Fig. 5g). Given this, we sought to show that PD-L1 on GLPMs and not other macrophages promoted tumor growth. We took peritoneal macrophages from PD-L1 KO mice or wild-type mice and transferred the cells into the peritoneum of Mac-Gata6 KO mice prior to inducing B16F10 liver metastasis. GLPMs from PD-L1-deficient mice had significantly decreased tumor burden compared to GLPMs from wild-type mice (Fig. 5h). These data demonstrate that tumor growth is promoted by upregulation of PD-L1 on GLPMs.

Exploring the interaction of GLPMs with other cells revealed frequent interactions with CD8^+^ T cells (Supplementary Fig. 4a). Some of these interactions were quite short in nature (less than 10 min) but the majority lasted 40–70 min (Supplementary Fig. 4a). Depletion of CD8^+^ T cells (Supplementary Fig. 4b) revealed that the killing of tumors induced by blocking PD-L1 was lost (Fig. 5i) implicating these CD8^+^ T cells in the benefit unleashed by immunotherapy.

### GLPM recruitment is a common mechanism in liver metastases with potential clinical significance

**Fig. 6:**
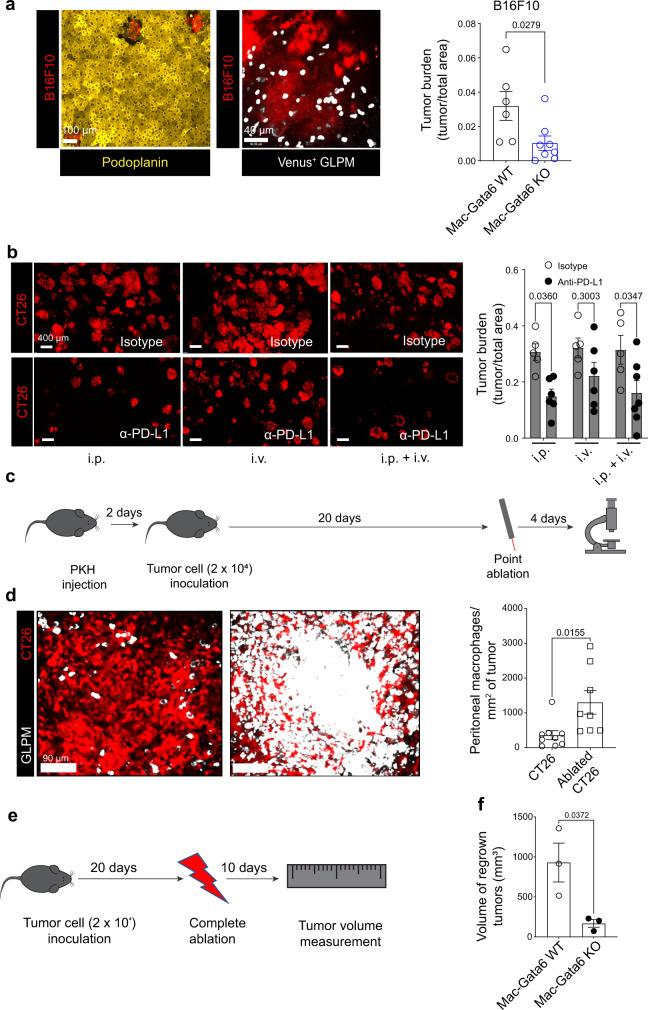
GLPM recruitment is a common mechanism in liver metastases with potential clinical significance. **a** Representative intravital images showing the mesothelium (yellow) and B16F10 metastases (red) bearing liver (left; scale bar = 100 µm), intravital image showing Gata6-Venus^+^ GLPMs (white) in the B16F10 metastases (red) (scale bar = 40 µm), and quantification of tumor burden in B16F10 metastases bearing liver of Mac-Gata6 WT vs Mac-Gata6 KO mice (right) (*n* = 6 for Mac-Gata6 WT, *n* = 8 for Mac-Gata6 KO), *P* values were calculated using two-tailed unpaired *t* test, *P* = 0.0279. **b** Representative intravital images (left; scale bar = 400 μm) and quantifications (right) showing CT26 tumor (red) burden in the liver of wild-type mice treated with intravenously and/or intraperitoneally anti-PD-L1 blocking antibody (*n* = 5 for isotype i.p., *n* = 6 for anti-PD-L1 i.p., *n* = 5 for isotype i.v., *n* = 6 for i.v. anti-PD-L1, *n* = 5 for isotype i.p. + i.v., *n* = 7 for anti-PD-L1 i.p. + i.v.), *P* values were calculated using an Ordinary two-way ANOVA with Bonferroni’s multiple comparisons test, *P* = 0.0360 i.p. isotype vs anti-PD-L1, *P* = 0.3003 i.v. isotype vs anti-PD-L1, *P* = 0.0347 i.p. + i.v. isotype vs anti-PD-L1. **c** Treatment regime for PKH, CT26 tumor cell inoculation and point ablation. **d** Representative intravital images (left; scale bar = 90 μm) and quantification (right) of GLPM localization in a growing CT26 liver metastasis (red) at the site of a point ablated liver metastasis (*n* = 9 for CT26, *n* = 8 for ablated CT26), *P* values were calculated using two-tailed unpaired *t* test, *P* = 0.0155. **e** Treatment regime of tumor cell model for measurement of tumor volume. **f** Quantification of regrown tumor volume after complete ablation of all the liver metastases in Mac-Gata6 WT and Mac-Gata6 KO mice (*n* = 3 per group), *p* values were calculated using two-tailed unpaired *t* test *P* = 0.0372. All graphs are presented as mean ± SEM. Source data are provided with this paper.

To ensure that GLPM recruitment is not a unique feature of CT26 liver metastases, we used B16F10 melanoma and 4T1 breast cancer cells in the same model of liver metastases. Both B16F10 and 4T1 liver metastases damaged the liver mesothelium and recruited GLPMs (Fig. 6a; Supplementary Fig. 5). Moreover, B16F10 tumor burden was reduced by 65% in Mac-Gata6 KO mice compared to Mac-Gata6 WT mice (Fig. 6a). This suggests that liver metastases-mediated mesothelial damage and GLPM recruitment is a common feature of various cancers that metastasize to the liver.

In our PD-L1 blocking experiments, the blocking antibody was given intraperitoneally to ensure it reached the GLPMs. This seemed intuitive at the time but became important from a therapeutic viewpoint. There is a big discrepancy between preclinical and clinical data regarding the efficacy of PD-1/PD-L1 checkpoint inhibitors in colorectal and other metastasis into liver^23,33,34^. In the rare studies where PD-L1 blocking antibody was tested against colorectal cancer metastasis growing in the liver, the blocking antibody was administered intraperitoneally whereas in clinical trials, anti-PD-1/PD-L1 checkpoint inhibitors are administered intravenously^23,33,34^. We administered PD-L1 blocking antibody either intraperitoneally, intravenously or both at 1 day following tumor cell inoculation and at every 3 days thereafter. Intraperitoneally administered PD-L1 blocking antibody significantly (52%) reduced tumor growth whereas intravenously administered PD-L1 blocking antibody was less effective (30%) in reducing tumor growth (Fig. 6b). Administration of PD-L1 blocking antibody via both routes did not provide any further benefits (Fig. 6b). Clearly, the clinically accepted, intravenous route of administration of anti-PD-L1 was not very effective in impacting the growth of liver metastases.

Tumor ablation is another approach of treating liver metastases although re-lapse is not uncommon perhaps due to incomplete ablation. To determine whether GLPMs could potentially help the tumor regrow, we used the modified protocol of inoculating only 20,000 tumor cells to get fewer but visible metastases (Fig. 6c). Twenty days after, thermal ablation was performed on all of the metastases in the liver. GLPM recruitment was imaged on day 4 after point ablation (Fig. 6d) and the volume of the regrown tumors was measured on day 10 after complete ablation (Fig. 6e). Thermal ablation caused a massive recruitment of GLPMs (Fig. 6d), far more than was seen in the initial tumors. The ablated tumors regrew at an alarming rate which could be reduced in Mac-Gata6 KO mice (Fig. 6f).

## Discussion

It is well established that monocytes infiltrate the tumor microenvironment to become macrophages that are skewed towards a “repair” like phenotype^5,6,17,35^. There is some debate with respect to the importance of monocyte-derived macrophage in liver metastasis of colorectal cancer origin^36^. Our own work suggests that these monocytes are only recruited into the tumor microenvironment if the tumor cells can produce and release monocyte specific chemokines such as CCL2. There is also growing evidence that tissue macrophages already present in organs can migrate to tumors and also assume a “repair” like phenotype^9,10^. However, this would seem less likely in liver where the Kupffer cells (macrophages) are immobilized in the vasculature and do not migrate towards these metastases^7^. To date, the millions of Gata6^+^ cavity macrophages found in the peritoneum, the pleura and the pericardium seem not to have drawn the attention of the cancer field for their ability to infiltrate tumors. In this study, we found that GLPMs are pro-tumorigenic by infiltrating the metastatic tumor microenvironment and interacting with CD8^+^ T cells via the PD-1/PD-L1 pathway. However, the tumor had to breach the mesothelium to be detected by the GLPMs via an ATP-dependent event. Recent work suggests that these Gata6^+^ cavity macrophages patrol the organs within the peritoneal and pericardial space and rapidly infiltrate when tissue injury occurs^14,15^. This is consistent with our data that the breach of the mesothelium injured some host cells and released ATP to attract these macrophages. Moreover, some necrotic cancer cells that are usually found in the core of growing tumors could be a secondary source of ATP in our system. It is worth mentioning that this is definitely not a mouse phenomenon as liver metastases can grow in the subcapsular region, in exophytic manner as well as within the liver parenchyma away from the capsule in humans^37,38^. Sensing growing metastases inside the liver is seemingly a difficult proposition for the GLPMs when the liver mesothelium remains intact. Interestingly, in vitro observations of ovarian cancer spheroids revealed clearance of cultured mesothelial monolayers^39^. Indeed, in ovarian cancer, mesothelial clearance is considered to be the first step in ovarian cancer peritoneal metastasis and whether GLPMs also contribute in this scenario would be worth examining.

Our data also suggest that treatment of these tumors would require an alternate mode of intervention, namely intraperitoneal administration. Indeed, we found better efficacy of checkpoint inhibitors in reducing tumor burden if they were administered intraperitoneally but not intravenously (standard of care). Interestingly, it is known that antibiotic administration is more efficacious if given via the peritoneal route to target peritoneal infections in the case of for example peritoneal dialysis and as such this route may also be amenable for cancer treatment^40,41^. It is quite interesting that PD-1-PD-L1 blockade works quite well in preclinical models of liver metastases^33^, but the same treatment fares poorly in patients with liver metastases^23,34^. There may be various reasons underlying this discrepancy but the apparent differences in the route of administration should be considered.

Surgical resection is considered as the gold standard in treating liver metastasis. However, only a small percentage of patients qualify for surgical resection^42^. Tumor ablation is becoming increasingly popular to destroy unresectable liver cancers of both primary and metastatic origins. Mostly a hot probe (radio frequency or microwave) or in some cases freezing (liquid nitrogen) is used to ablate a tumor^43^. Tumor ablation is considered to be very safe^44^; however, it’s efficacy in long-term control of colorectal cancer liver metastasis has been debated^45,46^. Local recurrence of tumor is a significant issue with tumor ablation^44,47,48^. Less than optimal ablation could be responsible for local recurrence of the tumor and as proof of principle in our mouse model, ablation attracted GLPMs in unprecedented numbers leading to tumor regrowth. In retrospect, this observation should not be too surprising given that these GLPMs are key for tissue healing. Indeed, the GLPMs dramatically increased the rate of regrowth in the ablated tumors. While our model does not necessarily mirror what is done clinically with liver tumors that are growing in the subcapsular region or exophytically, our study provides a proof of concept that depletion of GLPMs may reduce local recurrence of liver metastases. Indeed, our findings may provide alternate avenues in the fight against liver metastases and their recurrence by manipulating GLPMs and considering the peritoneal cavity as a portal for more effective drug delivery.

## Methods

### Mice

All experiments involving animals were approved by the University of Calgary Animal Care Committee under protocol AC20-0016 and followed guidelines established by the Canadian Council for Animal Care. All mice were housed under a 12/12 light/dark cycle at 22–25 °C and 30–70% humidity under specific pathogen-free conditions. Mice received sterilized rodent choaw and water ad libitum. C57BL/6J and BALB/c mice were purchased from the Jackson Laboratory. Gata6^fl/fl^ mice were kindly provided by Dr. Medzhitov (Yale University)^24^ and bred in-house with Lyz2^cre^ mice. Lyz2^cre^; Gata6^fl/fl^ mice were subsequently bred with Gata6^fl/fl^ to generate Cre+ (denoted as Mac-Gata6 KO) and Cre− (denoted as Mac-Gata6 WT) littermates. CCR2 KO (Ccr2^Rfp/Rfp^) mice were kindly provided by Richard M Ransohoff (Lerner Research Institute, Cleveland Clinic, Cleveland) and Israel F. Charo (University of California San Francisco, San Francisco)^49^. Gata6^H2B-Venus^ reporter mice were kindly provided by Dr. Hadjantonakis (Memorial Sloan Kettering)^50^. Lyz2^cre^; Gata6^fl/fl^, Gata6^fl/fl^ & Gata6^H2B-Venus^ mice were back-crossed onto BALB/c background for at least 8 generations to achieve greater than 98% pure background. The PD-L1 KO mice were kindly provided by Dr. Sharpe (Harvard Medical School). Single Nucleotide Polymorphisms (SNPs) testing was performed by Taconic Biosciences to confirm genetic purity. 8–12-week-old male and female mice were used for experiments. Mice were maintained in a specific pathogen-free facility at the University of Calgary Animal Resource Centre.

### Cell culture

Cancer (CT26 colorectal, ATCC CRL-2638; MC38 colorectal, Kerafast ENH204-FP; B16F10 melanoma, ATCC CRL-6475; and 4T1 breast cancer, ATCC CRL-2539) cells were cultured using RPMI 1640 or DMEM complete medium according to the suppliers’ instructions. The complete media were prepared by supplementing the basal media (Invitrogen) with 10% heat-inactivated FBS, L-glutamine, penicillin (100units/ml) and streptomycin (100 mg/ml). CT26, B16F10 and 4T1 cells that are stably transfected with iRFP were cultured in Puromycin selection medium. Cells were passaged or used for in vivo inoculation at 80% confluency. For single-cell suspension, tumor cells were detached with Puck’s EDTA solution (containing Potassium chloride, sodium chloride, sodium bicarbonate, dextrose, HEPES and EDTA) and washed in complete culture medium. CT26-iRFP cells were washed twice more with HBSS, viable cells were counted for inoculation. Before counting, MC38 cells were stained with DiD according to the manufacturer’s protocol. The ATCC Universal Mycoplasma Detection Kit was used to ensure the absence of Mycoplasma in all of the cell lines used.

Resident peritoneal macrophages were isolated by lavaging the peritoneum with 5 ml cold PBS. Isolated peritoneal macrophages were allowed to adhere onto cell culture treated petri dish. After 2 h, cells were washed three times with warm PBS to get rid of non-macrophage cells. Adherent macrophages were then cultured in complete RPMI for the apoptotic body co-incubation assay.

### In vivo interventions

#### Tumor cell inoculation

CT26-iRFP, B16F10-iRFP, 4T1-iRFP and DiD-stained MC38 cells were prepared as described before^51^. Briefly, a small incision was made on the upper left flank of the mouse and the spleen was exteriorized. 2 × 10^5^ or 2 × 10^4^ cells, as indicated in the figures, were intrasplenically inoculated, the spleen was removed within 1 min after inoculation to allow tumor cells to circulate to the liver and to avoid tumor growth in the spleen. Subsequently, the peritoneum was sutured, the skin was closed using staples and the mouse was injected with Buprenorphine (0.05 mg/kg; s.c.) to manage pain. CT26 and 4T1 cells were inoculated in mice of BALB/c background whereas MC38 and B16F10 cells were inoculated in mice of C57BL/6J background. Mice were euthanized upon experimental endpoint. Mice reached a humane endpoint and were humanely euthanized upon tumor sizes of 2 cm^2^ or larger, >20% body weight loss, hunched and isolated posture, or reluctance to move in response to gentle stimulation as approved by University of Calgary Animal Care Committee protocol no: AC20-0016.

#### Tumor ablation and mechanical disruption of mesothelium

For the tumor ablation experiments, 2 × 10^4^ CT26-iRFP cells were inoculated as described above and the tumors were allowed to grow for 20 days. This protocol allows the growth of fewer (3–9) and visible metastatic tumors in the liver. At day 20, the peritoneum was opened, the whole liver was exteriorized and all the tumors were point ablated using a hot needle or completely ablated using a cautery as such there was no visible tumors after complete ablation. Then, the peritoneum was sutured, the skin was closed using staples and the animal was injected with Buprenorphine (0.05 mg/kg; s.c.) to manage pain. On day 4 post point ablation, the ablation sites were imaged to visualize peritoneal GLPM recruitment. For the complete ablation study, the volume of the regrown tumors was measured using a Vernier caliper 10 days after ablation. Mac-Gata6 WT vs Mac-Gata6 KO mice were used to compare the volume of the regrown tumors.

For mechanical disruption of the mesothelium, a cell scraper was used to gently scrape the liver. Two days after mechanical disruption, intravital imaging was performed. This minor disruption heals within a week and the GLPMs do not stay in the liver when the disrupted mesothelium heals.

#### Generation of bone marrow chimeras

Bone marrow chimeric mice were generated by bone marrow transplantation using a standard protocol as previously described^52^. Briefly, wild-type BALB/c or C57BL/6J mice were lethally irradiated (2x 525cGY) and subsequently reconstituted with bone marrow cells from *Gata6*^*H2B-Venus*^ mice of appropriate background for 8 weeks.

### In vivo treatment

For blocking ATP receptor, mice received 10 µM P2RX7 antagonist (Tocris) or vehicle (saline) intraperitoneally starting from 24 h after tumor cell inoculation and continued every 24 h during the experiment. Anti-PD-L1 monoclonal antibody (clone: 10F.9G2; Bio X Cell) or Rat IgG2b, κ isotype control antibody at 10 mg/kg was administered via indicated routes starting from 24 h after tumor cell inoculation and continued every three day throughout the study period.

For CCL2 blocking experiments, CCL2 neutralizing antibody (clone: 2H5; Bio X Cell) intraperitoneally injected at 2 mg/kg starting from 24 h after tumor cell inoculation and every 3 days until the experimental endpoint.

### Peritoneal macrophage and CD8^+^ T cell depletion

Clodronate liposome and PBS liposome were purchased from clodronateliposomes.org (Vrije Universiteit, Netherlands). Peritoneal macrophage depletion was performed by intraperitoneal administration of 50 µL clodronate liposome/mouse 7 days prior to tumor cell inoculation. This treatment depletes resident peritoneal macrophages for more than three weeks. Mice treated with PBS liposome were used as control. CD8^+^ T cells were depleted before tumor cell inoculation by intraperitoneal administration of anti-CD8α (clone: 2.43; Bio X Cell) antibody at 4 day (400 µg/mouse) and 1 day (200 µg/mouse) before tumor cell inoculation. To maintain CD8^+^ T cell depletion, anti-CD8α antibody treatment was continued every three days (at 200 µg/mouse) during the study period. Rat IgG2b, κ isotype control antibody was used in the control group at the same dosage. CD8^+^ T cell depletion was verified using both anti-CD8β and anti-CD8α (clone: 53–6.7) antibodies.

### Peritoneal macrophage transfer

The peritoneal cells from PD-L1 deficient or wild-type mice were harvested with a peritoneal lavage as previously described^15^. All harvested cells were then directly transferred into Gata6 deficient mice via intraperitoneal injection at 7 days before tumor cell inoculation

### Spinning disc confocal intravital microscopy

A tail vein catheter was inserted into mice after anesthetization with 200 mg/kg ketamine (Bayer Animal Health) and 10 mg/kg xylazine (Bimeda-MTC). Surgical preparation of the liver intravital imaging was performed by anesthetizing mice and surgically exteriorizing the liver^53^. Image acquisition was performed using Olympus IX81 inverted microscope, equipped with an Olympus focus drive and a motorized stage (Applied Scientific Instrumentation, Eugene, OR) and fitted with a motorized objective turret equipped with 4 ×/0.16 UPLANSAPO, 10 × /0.40 UPLANSAPO and 20× /0.70 UPLANSAPO objective lenses and coupled to a confocal light path (WaveFx; Quorum Technologies, Guelph, ON) based on a modified Yokogawa CSU-10 head (Yokogawa Electric Corporation, Tokyo, Japan). Peritoneal macrophages were selectively and specifically labeled by intraperitoneal administration of a special formulation of PKH26 dye that can be taken up only by phagocytes as described elsewhere^54^ two days before tumor cell inoculation. For experiments labeling Kupffer cells, fluorescent F4/80 antibody was administered intravenously to label any intravascular macrophages, and not GLPMs. Target cells within the liver were visualized using fluorescently labeled antibodies. Laser excitation wavelengths 491-, 561-, and 642-nm (Cobolt) were used in a rapid succession together with the appropriate band-pass filters (Semrock). A back-thinned EMCCD 512 × 512 pixel camera was used for fluorescence detection. Volocity software (Perkin Elmer) was used to drive the confocal microscope and to analyze the images.

### Quantification of tumor burden

CT26-iRFP and B16F10-iRFP liver metastases were quantified at indicated time points as described previously^51^. Briefly, single images (×10 lens) were recorded with an electronic computer-controlled stage and subsequently stitched together (10% overlap for each image to obtain an overview image of >20 mm^2^ of the liver, called a “stitched image”. Tumor area within each stitched image was quantified using Volocity software and tumor burden (tumor area/total area imaged) was calculated.

### Cell isolation and flow cytometry

For isolating non-parenchymal cells from normal and metastases bearing livers a previously described protocol was used^55^. Briefly, livers were perfused with HBSS, minced into small pieces digested in collagenases. Single-cell suspensions were generated by mechanical disruption through a 70-µm nylon mesh (BD Bioscience). Cellular debris and hepatocytes were removed by 33% isotonic Percoll (Sigma-Aldrich). Single-cell suspensions from peritoneal lavage were collected as described^11^. Dead cells were excluded using fixable viability dye (eBioscience). Cell surface-expressed molecules were stained on ice for 20–30 min^15^. Intracellular and nuclear staining was performed by using the Foxp3 nuclear factor staining buffer set (eBioscience). Antibodies used for flow cytometry are included in Supplementary Table 1. The samples were run using a BD FACS Canto Cytometer (Life Technologies) and analyzed using FlowJo (Tree Star).

### Multiplex cytokine assay

CT26, B16F10 and MC38 cells were cultured as described above for 24 h, the medium was collected and centrifuged at 500 × *g* for 10 min. The supernatant was transferred into a separate centrifugal filter unit (Milipore, 0.22 μm, Durapore-PVDF). The flow through was collected after centrifugation. Then 30 μl of each sample was used to measure cytokines with the MILLIPLEX^®^MAP Kit (MYCTOMAG-70K-PMX). The preparation was done according to the supplier’s protocol provided with the kit. The plate was analyzed using a Luminex 200 ™ device.

### Isolation of apoptotic bodies

Apoptotic bodies were isolated as described previously^56^. Briefly, CT26-iRFP cells were grown to confluence, the medium was collected in a 50-ml tubes, centrifuged at 500 × *g* for 5 min to remove dead cells. The supernatant was centrifuged at 2000 × *g* for 20 min to collect the apoptotic bodies. Apoptotic bodies were washed once with cold HBSS, resuspended in complete medium and co-incubated with resident peritoneal macrophages in a 10-cm^2^ cell culture treated petri dish for 15 h.

### Statistics

All data are presented as mean ± SEM. Statistical analyses were performed using GraphPad Prism software. Unpaired two- tailed t test, one-way, or two-way ANOVA followed by Tukey’s post hoc test for multiple comparisons were used to compare different groups. Survival curves were compared using log-rank (Mantel-Cox) test. Statistical significance was set at *p* < 0.05.

### Reporting summary

Further information on research design is available in the Nature Research Reporting Summary linked to this article.

## Supplementary information

Supplementary information

Description of Additional Supplementary Files

Supplementary movie 1

Supplementary movie 2

Supplementary movie 3

Reporting Summary

## Data Availability

All data are available within the Article, Supplementary Information or Source Data file. Source data are provided with this paper.

## Source data

Source Data
