# Supplementary material for: Gata6+ resident peritoneal macrophages promote the growth of liver metastasis
Source: Nat Commun. 2022 Jul 29;13:4406. doi: 10.1038/s41467-022-32080-y (PMC9338095; doi:10.1038/s41467-022-32080-y)
Supplement: Supplementary file 1 — Supplementary information [file 41467_2022_32080_MOESM1_ESM.pdf]

SUPPLEMENTARY INFORMATION

A source of Gata6^{+} resident peritoneal macrophages promote the growth of liver metastasis

Mokarram Hossain[1,3#], Raymond Shim[1,3#], Woo-Yong Lee[3], Arlene H. Sharpe[4,5,6], Paul Kubes[1,2,3,*]

[1]Department of Physiology and Pharmacology, [2]Microbiology and Immunology, [3]Snyder Institute for Chronic Diseases, University of Calgary, 3330 Hospital Drive NW, Calgary, Alberta, T2N 4N1.

[4]Department of Immunology, Blavatnik Institute, Harvard Medical School, Harvard Medical School and Brigham and Women's Hospital, Boston, MA 02115, USA.

[5]Broad Institute of MIT and Harvard, Cambridge, MA 02142, USA.

[6]Department of Pathology, Brigham and Women's Hospital, Boston, MA 02115, USA.

*Correspondence: pkubes@ucalgary.ca

[#] These authors contributed equally

Supplementary figures

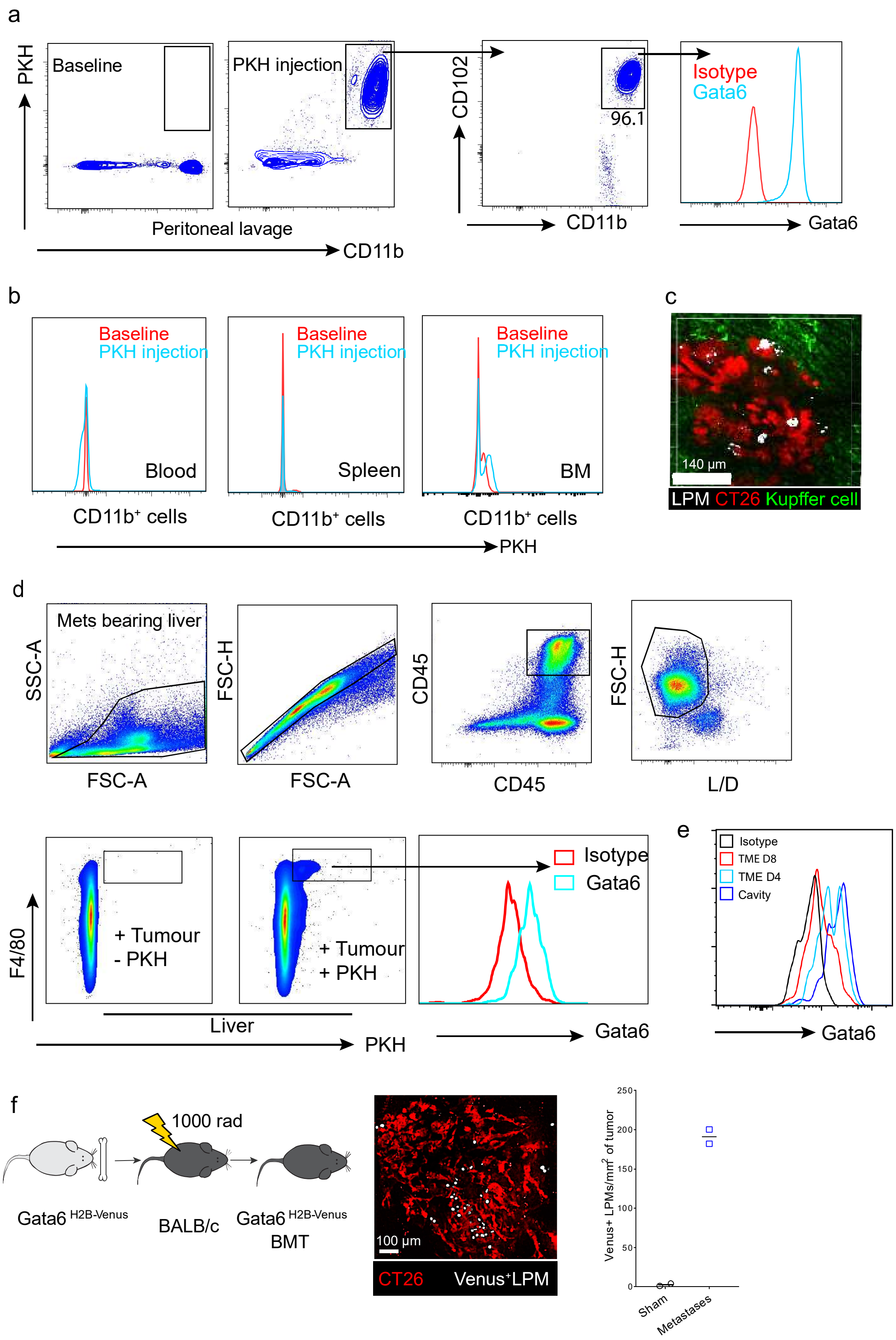
a
PKH
Baseline
PKH injection
Peritoneal lavage
CD11b
CD102
96.1
CD11b
Isotype
Gata6
Gata6
b
Baseline
PKH injection
Blood
Spleen
BM
CD11b+ cells
CD11b+ cells
CD11b+ cells
PKH
c
140 µm
LPM CT26 Kupffer cell
d
Mets bearing liver
SSC-A
FSC-A
FSC-H
FSC-A
CD45
CD45
FSC-H
L/D
F4/80
+ Tumour
- PKH
+ Tumour
+ PKH
Liver
PKH
Isotype
Gata6
Gata6
e
Isotype
TME D8
TME D4
Cavity
Gata6
f
1000 rad
Gata6 H2B-Venus
BALB/c
Gata6 H2B-Venus
BMT
100 µm
CT26 Venus+LPM
Venus+ LPMs/mm2 of tumor
250
200
150
100
50
0
Sham
Metastases

Supplementary Figure 1. GLPMs in CT26 liver metastases. **a** Representative flow cytometry plots showing specific PKH labeling of GLPMs, and **b** leaving myeloid cells of blood, spleen and bone marrow origin unstained (bottom row). **c** Stitched intravital image (scale bar = 140 μm) showing PKH^{+} GLPMs (white) in CT26 liver metastasis (red). Kupffer cells (bright green) around the metastasis are not labeled with PKH. **d** Representative flow cytometry plots showing gating strategy for identifying GLPMs from the metastases bearing livers where PKH^{+} macrophages in the TME express GLPM-specific transcription factor Gata6. **e** Representative histogram plot of Gata6 expression of GLPMs from the peritoneal cavity or GLPMs in the TME at 4 and 8 days after tumor cell inoculation (n = 3; from one independent experiment). **f** Generation of $Gata6^{H2B\text{-}Venus}$ reporter mice following irradiation and bone marrow transfer into BALB/c mice (left), representative images of the $Gata6^{H2B\text{-}Venus}$ GLPMs (white) following CT26 liver metastases (red) (middle), and quantification of GLPMs on CT26 liver metastases (right; n = 2 per group), data is presented as mean.

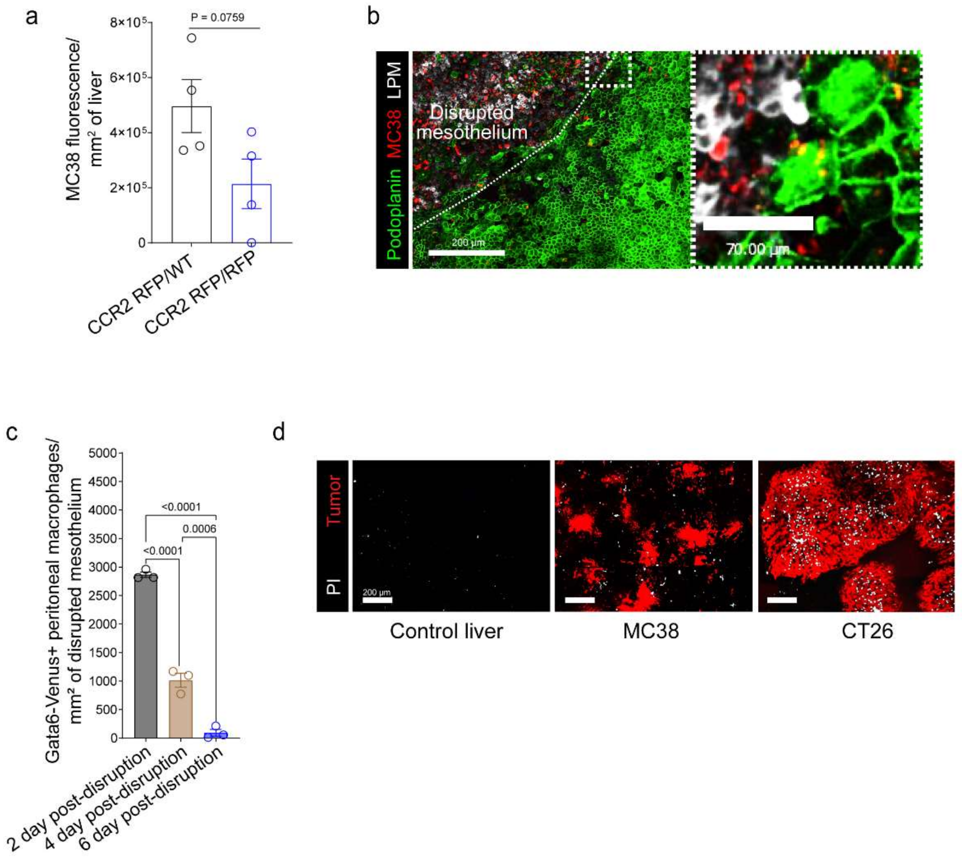

Supplementary Figure 2. Mesothelium disruption is required for GLPM recruitment to liver metastases. a Quantification of MC38 fluorescence per mm^2 of liver (n = 4 per group), *P* values were calculated using two-tailed unpaired t-test, *P* = 0.0759. **b** Intravital image (left; scale bar = 200 µm) and a magnified image of the selected area (right; scale bar = 70 µm) showing GLPM (white) recruitment in MC38 metastases (red) bearing liver where the mesothelium (podoplanin; green) is mechanically disrupted. **c** Quantification of Gata6-Venus$^+$ peritoneal macrophage on the disrupted mesothelium at 2-, 4- and 6-days post-disruption (n = 3 per group) *P* values were calculated using an Ordinary one-way ANOVA with Tukey's multiple comparisons test, *P* < 0.0001 2 day vs 4 day or 6 day post-disruption, *P* = 0.0006 4 day vs 6 day post-disruption. **d** Representative intravital images (scale bar = 200 µm) showing PI$^+$ cells (white) in control liver, MC38 metastases (red) or CT26 metastases (red) bearing liver (n = 3; from one independent experiment). All graphs are presented as mean ± SEM.

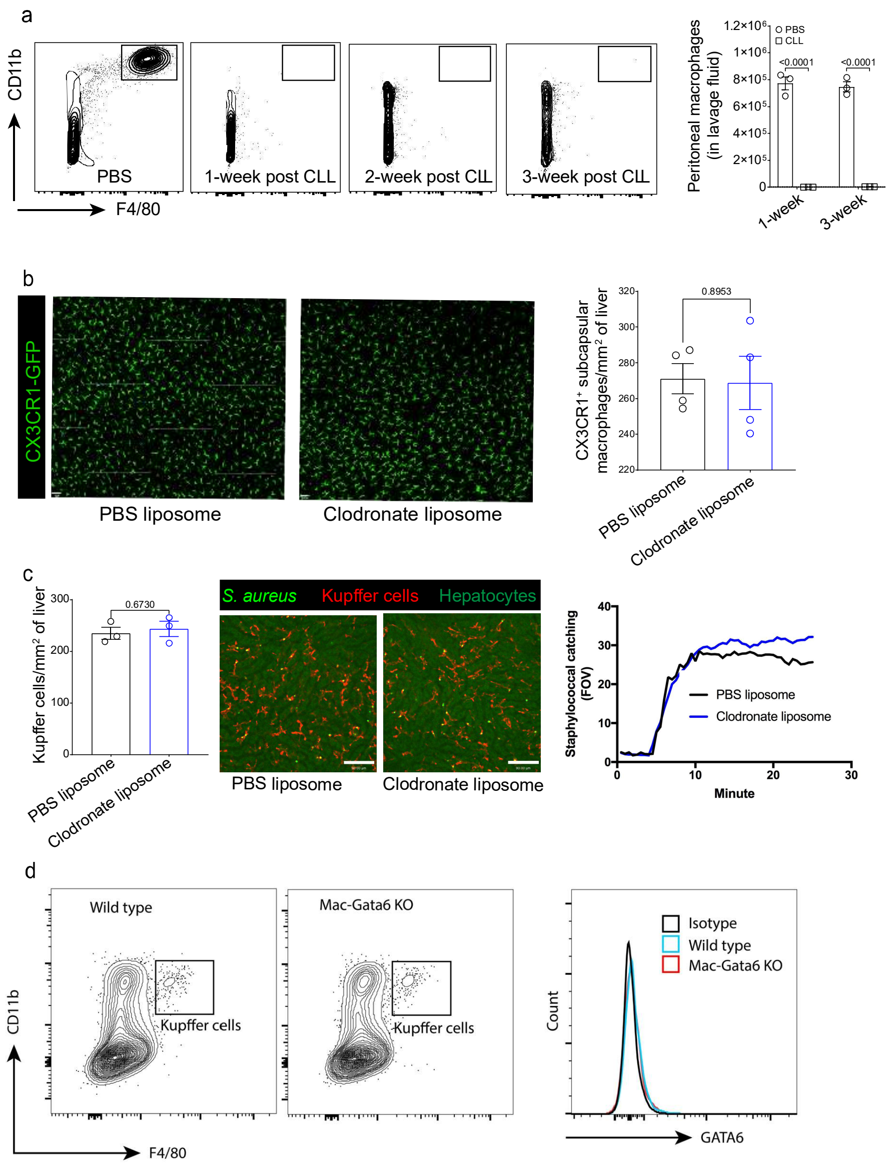

Supplementary Figure 3. Liver macrophage populations are not altered at 7-days after Clodronate loaded liposomes treatment. a Representative flow cytometry plots (left) and quantification (right) showing efficacy of peritoneal macrophage depletion following 50 μl of CLL over 3 weeks (n = 3 per group), *P* values were calculated using an Ordinary two-way ANOVA with Bonferroni's multiple comparisons test, $P < 0.0001$ PBS vs CLL. **b** Representative intravital images (left) and quantification (right) of CX3CR1-GFP macrophages (green) following PBS liposome or CLL treatment (n = 4 per group) *P* values

were calculated using two-tailed unpaired t-test, $P = 0.8953$. **c** Quantification of Kupffer cells in the liver after PBS liposome or CLL treatment (n = 3 per group) P values were calculated using two-tailed unpaired t-test, $P = 0.6730$ (left) and representative intravital images (middle) and quantification (right) of Kupffer cell (red) catching of *S. aureus* (bright green) following PBS liposome or CLL treatment. **d** Representative flow cytometry plots and histogram showing lack of Gata6 expression in Kupffer cells in both WT and Mac-Gata6 KO mice (n = 3; from one independent experiment). All graphs are presented as mean ± SEM.

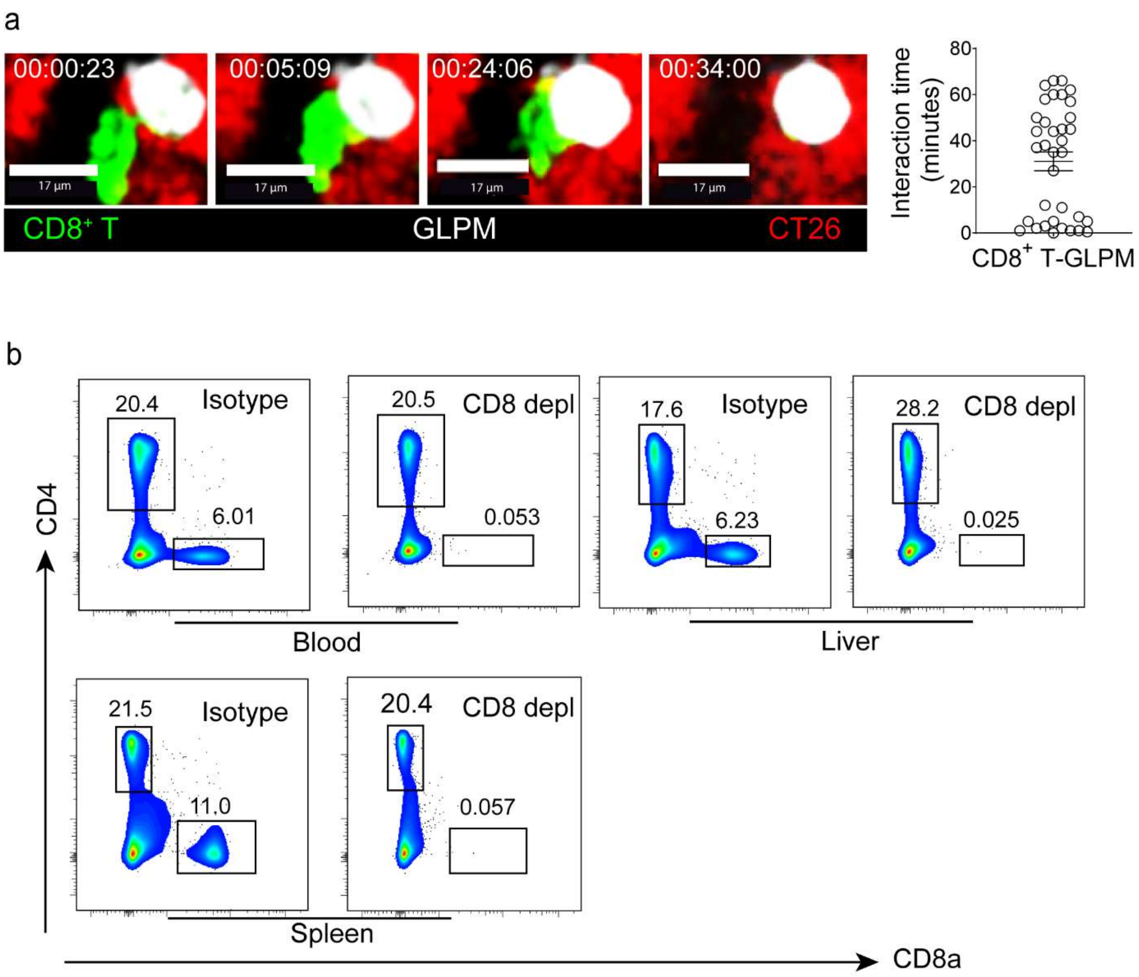

Supplementary Figure 4. CD8+ T cell role in liver metastases. a Time-lapse intravital images (left; scale bar = 17 µm) showing interaction between a GLPM (white) and a CD8+ T cell (green) within CT26 metastasis (red) and the quantification (right) of the interaction time between GLPMs and CD8+ T cells within the TME (each point indicates an interaction; n=3). **b** Representative flow cytometry plots showing efficiency of CD8+ T cell depletion in the blood, liver and spleen following intraperitoneal administration of anti-CD8α (clone: 2.43; Bio X Cell) antibody (n = 5; from one independent experiment). All graphs are presented as mean ± SEM.

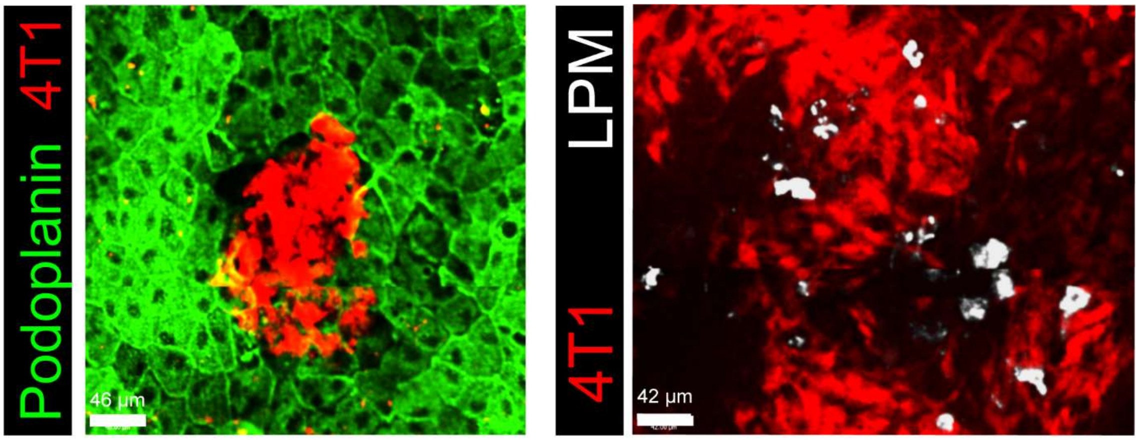

Supplementary Figure 5. Mesothelial damage and GLPM recruitment in 4T1 liver metastasis. Representative confocal images (left; scale bar = 46 µm) displaying the mesothelium (green) and 4T1 metastasis (red) bearing liver and (right; scale bar = 42 µm) PKH$^+$ GLPMs (white) in 4T1 metastasis (red).

Supplementary Table 1. Table of all antibodies used for flow cytometry.

**Fluorophore**	**Antibody**	**Clone**	**Supplier**	**cat no**	**lot no**	**dilution**
AF647	Hamster anti-mouse podoplanin	eBio8.1.1	eBioscience	53-5381-82	B334381	1:100
AF647	Rat anti-mouse CD8b	Ly-3	Biolegend	126612	B204556	1:100
APC	Rat anti-mouse PD-1	29F.1A12	Biolegend	135209	B2202264	1:100
APC	Rat IgG2a (isotype control)	RTK2758	Biolegend	400511	B207074	1:100
APC	Rat anti-mouse MHCII	M5/114.15.2	BD Biosciences	562367	8310917	1:100
APC Cy7	Rat anti-mouse F4/80	BM8	eBioscience	47-4801-82	2086942	1:100
BV510	Rat anti-mouse CD45	30-F11	Biolegend	103138	B346257	1:100

BV605	Rat anti-mouse CD102	3C4(m1c2/4)	BD Biosciences	740346	233587	1:100
eFluor450	Hamster anti-mouse CD3	145-2C11	ThermoFisher	48-0031-82	1993648	1:100
eFluor450	Anti-mouse F4/80	BM8	eBioscience	48-4801-80	1974936	1:100
FITC	Rat anti-mouse F4/80	BM8	eBioscience	11-4801-85	2B330107	1:100
Pacific blue	Rat anti-mouse Ly6G	1A8	Biolegend	127612	B336505	1:100
PE	Rat anti-mouse CD8b	YTS156.7.7	Biolegend	126608		1:100
PE	Rat anti-mouse PD-L1	10F9G2	Biolegend	124307		1:100
PE	Rat IgG2b (Isotype control)	RTK4530	Biolegend	400607		1:100

PerCp Cy5.5	Rat anti-mouse Ly6C	HK1.4	Biolegend	128012	B338815	1:100
PE Cy7	Rat anti-mouse CD11b	M1/70	ThermoFisher	25-0112-82	2394478	1:100
PE	Rat anti-mouse CD4	RM4-5	eBioscience	12-0043082	E01014-1633	1:100
FITC	Rat anti-mouse CD8	53-6.7	eBioscience	11-0081-85	2202710	1:100
FITC	Hamster anti-mouse CD80	16-10A1	BD Pharmingen	553768	7493	1:100
Unconjugated	Rat anti0mouse CD16/32	BE307	BioXcell	BE0307		1:4000
