# Supplementary material for: Gata6+ resident peritoneal macrophages promote the growth of liver metastasis
Source: Nat Commun. 2022 Jul 29;13:4406. doi: 10.1038/s41467-022-32080-y (PMC9338095; doi:10.1038/s41467-022-32080-y)
Supplement: Supplementary file 2 — Description of Additional Supplementary Files [file 41467_2022_32080_MOESM2_ESM.pdf]

Supplementary movie 1. 3D image of localization of GLPMs (white) in respect to CT26 liver metastasis (red). Scale bar = 15 μm.

Supplementary movie 2. 3D representation of PI (red) colocalising with podoplanin (green) cells following CT26 liver metastases (purple).

Supplementary movie 3. In vivo visualisation of uptake of tumor particle (red) by GLPM (green). Scale bar = 15 μm
