# Supplementary material for: Gata6+ resident peritoneal macrophages promote the growth of liver metastasis
Source: Nat Commun. 2022 Jul 29;13:4406. doi: 10.1038/s41467-022-32080-y (PMC9338095; doi:10.1038/s41467-022-32080-y)
Supplement: Supplementary file 6 — Reporting Summary [file 41467_2022_32080_MOESM6_ESM.pdf]

nature research

Corresponding author(s): Dr. Paul Kubes

Last updated by author(s): Jul 14, 2022

Reporting Summary

Nature Research wishes to improve the reproducibility of the work that we publish. This form provides structure for consistency and transparency in reporting. For further information on Nature Research policies, see our Editorial Policies and the Editorial Policy Checklist.

Statistics

For all statistical analyses, confirm that the following items are present in the figure legend, table legend, main text, or Methods section.

n/a	Confirmed	
☐	☒	The exact sample size (n) for each experimental group/condition, given as a discrete number and unit of measurement
☐	☒	A statement on whether measurements were taken from distinct samples or whether the same sample was measured repeatedly
☐	☒	The statistical test(s) used AND whether they are one- or two-sided<br>*Only common tests should be described solely by name; describe more complex techniques in the Methods section.*
☒	☐	A description of all covariates tested
☒	☐	A description of any assumptions or corrections, such as tests of normality and adjustment for multiple comparisons
☐	☒	A full description of the statistical parameters including central tendency (e.g. means) or other basic estimates (e.g. regression coefficient) AND variation (e.g. standard deviation) or associated estimates of uncertainty (e.g. confidence intervals)
☐	☒	For null hypothesis testing, the test statistic (e.g. F, t, r) with confidence intervals, effect sizes, degrees of freedom and P value noted<br>*Give P values as exact values whenever suitable.*
☒	☐	For Bayesian analysis, information on the choice of priors and Markov chain Monte Carlo settings
☒	☐	For hierarchical and complex designs, identification of the appropriate level for tests and full reporting of outcomes
☒	☐	Estimates of effect sizes (e.g. Cohen's d, Pearson's r), indicating how they were calculated

Our web collection on statistics for biologists contains articles on many of the points above.

Software and code

Policy information about availability of computer code

Data collection	Images were acquired and quantified by Volocity software (Quorum Technologies) v6.1.1. Flow cytometry data were acquired with BD FACSDiva™ (BD Biosciences) v8.0.2
Data analysis	Flowjo (Treestar Inc) v10 was used to analyze Flow cytometry data. Statistical analysis was performed in Graphpad Prism 9 v9.0 software .

For manuscripts utilizing custom algorithms or software that are central to the research but not yet described in published literature, software must be made available to editors and reviewers. We strongly encourage code deposition in a community repository (e.g. GitHub). See the Nature Research guidelines for submitting code & software for further information.

Data

Policy information about availability of data

All manuscripts must include a data availability statement. This statement should provide the following information, where applicable:

- Accession codes, unique identifiers, or web links for publicly available datasets
- A list of figures that have associated raw data
- A description of any restrictions on data availability

All data are available within the Article Supplementary Information or Source Data file.

Field-specific reporting

For a reference copy of the document with all sections, see nature.com/documents/nr-reporting-summary-flat.pdf

Life sciences study design

All studies must disclose on these points even when the disclosure is negative.

Sample size	In most experiments sample size was determined based on previous studies within the lab using these techniques (at last three biological replicates for each group). For intravital microscopy, we were limited by imaging only one mouse at a time so a minimum of 1 experimental mouse and 1 control mouse was imaged per day. Sample size was determined based on prior studies and literature using similar experimental paradigms (Wang et al., Science, 2017; Zindel et al., Science, 2021; Neupane et al., Cell, 2020; Babes et al., Cancer Immunology Research, 2022).
Data exclusions	We did not exclude any data.
Replication	Most of the experiments were repeated at least once and the results were reproducible. Data are pooled from replicated experiments.
Randomization	Mice were randomly selected into different treatment groups. Randomization was stratified by genotype.
Blinding	The investigators were not blinded during data acquisition because treatments and data collection were performed by the same researcher. Quantification of data did not require any interpretation or subjective judgment so blinding was not necessary.

Reporting for specific materials, systems and methods

We require information from authors about some types of materials, experimental systems and methods used in many studies. Here, indicate whether each material, system or method listed is relevant to your study. If you are not sure if a list item applies to your research, read the appropriate section before selecting a response.

Materials & experimental systems

n/a	Involved in the study	
☐	☒	Antibodies
☐	☒	Eukaryotic cell lines
☒	☐	Palaeontology and archaeology
☐	☒	Animals and other organisms
☒	☐	Human research participants
☒	☐	Clinical data
☒	☐	Dual use research of concern

Methods

n/a	Involved in the study	
☒	☐	ChIP-seq
☐	☒	Flow cytometry
☒	☐	MRI-based neuroimaging

Antibodies

Antibodies used

Fluorophore/antibody / clone / supplier name / catalog # / lot #/ dilution
AF488 / Hamster anti-mouse podoplanin / eBio8.1.1 / eBioscience / Cat# 53-5381-82 / B334381 / 1:100
AF647 / goat anti-rabbit IgG / polyclonal / Life Technologies / Cat# A21244 / 2390713 / 1:100
AF647 / rat anti-mouse CD8b / Ly-3 / Biolegend / Cat# 126612 / B204556 /1:100
APC / rat anti-mouse PD-1 / 29F.1A12 / Biolegend / Cat# 135209 / B202264 / 1:100
APC / Rat IgG2a (Isotype control) / RTK2758 / Biolegend / Cat# 400511 / B207074 / 1:100
AAPC / rat anti-mouse MHCII / M5/114.15.2 / BD Biosciences / Cat# 562367 / 8310917 / 1:100
APC-Cy7 / rat anti-mouse F4/80 / BM8 / eBioscience / Cat# 47-4801-82 / 2086942 /1:100
BV510 / rat anti-mouse CD45 / 30-F11 / Biolegend / Cat# 103138 / B346257 /1:100
BV605 / rat anti-mouse CD102 / 3C4(m1c2/4) / BD Biosciences / Cat# 740346 / 233587 /1:100
eFluor450 / hamster anti-mouse CD3 / 145-2C11 / ThermoFisher / Cat# 48-0031-82 / 1993648 /1:100
eFluor450 / anti-mouse F4/80 / BM8 / eBioscience / Cat# 48-4801-80 / 1974936 /1:100
FITC / rat anti-mouse F4/80 / BM8 / eBioscience / Cat#11-4801-85 / 2B330107 /1:100
Pacific Blue / rat anti-mouse Ly6G / 1A8 / Biolegend / Cat# 127612 / B336505 /1:100
PE / rat anti-mouse CD8 / YTS156.7.7 / Biolegend / Cat# 126608 / 1:100
PE / rat anti-mouse PD-L1 / 10F.9G2 / Biolegend / Cat# 124307 / 1:100
PE / Rat IgG2b (isotype control) / RTK4530 / Biolegend / Cat# 400607 / 1:100
PerCP-Cy5.5 / rat anti-mouse Ly6C / HK1.4 / Biolegend / Cat# 128012 / B338815 / 1:100
PE-Cy7 / rat anti-mouse CD11b / M1/70 / ThermoFisher / Cat# 25-0112-82 / 2394478 / 1:100
PE / rat anti-mouse CD4 / RM4-5 / eBioscience / Cat# 12-0043-82 / E01014-1633 / 1:100
FITC / rat anti-mouse CD8 / 53-6.7 / eBioscience / Cat# 11-0081-85 / 2002710 / 1:100
FITC / hamster anti-mouse CD80 / 16-10A1 / Pharmingen / Cat# 553768/ 7493 /1:100
Unconjugated / rabbit anti-human Gata6 / D61E4 / Cell Signaling / Cat# 5851
Unconjugated / rabbit IgG (Isotype control) / DA1E / Cell Signaling / Cat#3900S
Unconjugated / rat anti-mouse CD16/32 / BE0307 / BioXcell / Cat# BE0307 / 1:4000
Unconjugated / rat anti-mouse PD-L1 / 10F.9G2 / BioXcell / Cat# BE0101 / 10 mg/kg

Unconjugated / rat anti-mouse CD8 / BE0061 / BioXcell / Cat# BE0061 / 732019A1B / 200 - 500 ug per mouse

Validation

- CD16/CD32 functional blocking antibody has been reported to block Fc receptors in vitro and in vivo. Ab validation at https://d2a7cdyquyl45u.cloudfront.net/tds-sheets/BE0307-tds.pdf
- PD-L1 functional blocking antibody has been reported to block PD-L1 in numerous published articles. Validation at https://d2a7cdyquyl45u.cloudfront.net/tds-sheets/BE0101-tds.pdf
- CD8 unconjugated (depleting) antibody has been reported to deplete CD8 T cells and the supporting data is presented in Supplementary Figure 3.
- For all the antibodies used to stain specific cell markers, a positive and negative counter-stain was defined to identify signal to background ratio at concentrations ranging from 1:100 to 1:1000.

Fluorophore/antibody / clone / supplier name / catalog # / validation statement
AF488 / Hamster anti-mouse podoplanin / eBio8.1.1 / eBioscience / Cat# 53-5381-82 / https://www.thermofisher.com/order/genome-database/dataSheetPdf?producttype=antibody&productsubtype=antibody_primary&productId=53-5381-82&version=233
AF647 / goat anti-rabbit IgG / polyclonal / Life Technologies / Cat# A21244 / https://www.thermofisher.com/order/genome-database/dataSheetPdf?producttype=antibody&productsubtype=antibody_secondary&productId=A-21244&version=233
AF647 / rat anti-mouse CD8b / Ly-3 / Biolegend / Cat# 126612 / https://www.biolegend.com/en-us/search-results/alexa-fluor-647-anti-mouse-cd8b-antibody-4478?pdf=true&displayInline=true&leftRightMargin=15&topBottomMargin=15&filename=Alexa%20Fluor%C2%AE%20647%20anti-mouse%20CD8b%20Antibody.pdf
APC / rat anti-mouse PD-1 / 29F.1A12 / Biolegend / Cat# 135209 / https://www.biolegend.com/fr-lu/products/apc-anti-mouse-cd279-pd-1-antibody-6497?pdf=true&displayInline=true&leftRightMargin=15&topBottomMargin=15&filename=APC%20anti-mouse%20CD279%20(PD-1)%20Antibody.pdf
APC / Rat IgG2a (Isotype control) / RTK2758 / Biolegend / Cat# 400511 / https://www.biolegend.com/it-it/products/apc-rat-igg2a-kappa-isotype-ctrl-1838?pdf=true&displayInline=true&leftRightMargin=15&topBottomMargin=15&filename=APC%20Rat%20IgG2a,%20%CE%BA%20Isotype%20Ctrl%20Antibody.pdf
APC / rat anti-mouse MHCII / M5/114.15.2 / BD Biosciences / Cat# 562367 / 8310917 / https://www.bdbiosciences.com/content/bdb/paths/generate-tds-document.ca.562367.pdf
APC-Cy7 / rat anti-mouse F4/80 / BM8 / eBioscience / Cat# 47-4801-82 / https://www.thermofisher.com/order/genome-database/dataSheetPdf?producttype=antibody&productsubtype=antibody_primary&productId=47-4801-82&version=233
BV510 / rat anti-mouse CD45 / 30-F11 / Biolegend / Cat# 103138 / https://www.biolegend.com/en-us/products/brilliant-violet-510-anti-mouse-cd45-antibody-7995?pdf=true&displayInline=true&leftRightMargin=15&topBottomMargin=15&filename=Brilliant%20Violet%20510%E2%84%A2%20anti-mouse%20CD45%20Antibody.pdf
BV605 / rat anti-mouse CD102 / 3C4(m1c2/4) / BD Biosciences / Cat# 740346 / https://www.bdbiosciences.com/content/bdb/paths/generate-tds-document.us..pdf
eFluor450 / hamster anti-mouse CD3 / 145-2C11 / ThermoFisher / Cat# 48-0031-82 / https://www.thermofisher.com/order/genome-database/dataSheetPdf?producttype=antibody&productsubtype=antibody_primary&productId=48-0031-82&version=233
eFluor450 / anti-mouse F4/80 / BM8 / eBioscience / Cat# 48-4801-80 / https://www.thermofisher.com/order/genome-database/dataSheetPdf?producttype=antibody&productsubtype=antibody_primary&productId=48-4801-82&version=233
FITC / rat anti-mouse F4/80 / BM8 / eBioscience / Cat#11-4801-85 / https://www.thermofisher.com/order/genome-database/dataSheetPdf?producttype=antibody&productsubtype=antibody_primary&productId=11-4801-82&version=233
Pacific Blue / rat anti-mouse Ly6G / 1A8 / Biolegend / Cat# 127612 / https://www.biolegend.com/nl-nl/search-results/pacific-blue-anti-mouse-ly-6g-antibody-6082?pdf=true&displayInline=true&leftRightMargin=15&topBottomMargin=15&filename=Pacific%20Blue%E2%84%A2%20anti-mouse%20Ly-6G%20Antibody.pdf
PE / rat anti-mouse CD8 / YTS156.7.7 / Biolegend / Cat# 126608 / https://www.biolegend.com/fr-ch/products/pe-anti-mouse-cd8b-antibody-4476?pdf=true&displayInline=true&leftRightMargin=15&topBottomMargin=15&filename=PE%20anti-mouse%20CD8b%20Antibody.pdf
PE / rat anti-mouse PD-L1 / 10F.9G2 / Biolegend / Cat# 124307 / https://www.biolegend.com/fr-ch/products/pe-anti-mouse-cd274-b7-h1-pd-l1-antibody-4497?pdf=true&displayInline=true&leftRightMargin=15&topBottomMargin=15&filename=PE%20anti-mouse%20CD274%20(B7-H1,%20PD-L1)%20Antibody.pdf
PE / Rat IgG2b (isotype control) / RTK4530 / Biolegend / Cat# 400607 / https://www.biolegend.com/fr-ch/products/pe-rat-igg2b-kappa-isotype-ctrl-1856?pdf=true&displayInline=true&leftRightMargin=15&topBottomMargin=15&filename=PE%20Rat%20IgG2b,%20%CE%BA%20Isotype%20Ctrl%20Antibody.pdf
PerCP-Cy5.5 / rat anti-mouse Ly6C / HK1.4 / Biolegend / Cat# 128012 / https://www.biolegend.com/ja-jp/products/percp-cyanine5-5-anti-mouse-ly-6c-antibody-5967?pdf=true&displayInline=true&leftRightMargin=15&topBottomMargin=15&filename=PerCP/Cyanine5.5%20anti-mouse%20Ly-6C%20Antibody.pdf
PE-Cy7 / rat anti-mouse CD11b / M1/70 / ThermoFisher / Cat# 25-0112-82 / https://www.thermofisher.com/order/genome-database/dataSheetPdf?producttype=antibody&productsubtype=antibody_primary&productId=25-0112-82&version=233
PE / rat anti-mouse CD4 / RM4-5 / eBioscience / Cat# 12-0043-82 / https://www.thermofisher.com/document-connect/document-connect.html?url=https://assets.thermofisher.com/TFS-Assets%2Fcertificate%2FCBD%2FCOA%2FCOA_12-0043-82_2345064_1.pdf
FITC / rat anti-mouse CD8 / 53-6.7 / eBioscience / Cat# 11-0081-85 / https://www.thermofisher.com/order/genome-database/dataSheetPdf?producttype=antibody&productsubtype=antibody_primary&productId=11-0081-82&version=233
FITC / hamster anti-mouse CD80 / 16-10A1 / BD Pharmingen / Cat# 553768/ https://www.bdbiosciences.com/content/bdb/paths/generate-tds-document.ca.553768.pdf
Unconjugated / rabbit anti-human Gata6 / D61E4 / Cell Signaling / Cat# 5851 / https://www.cellsignal.com/datasheet.jsp?productId=5851&images=1&size=A4
Unconjugated / rabbit IgG (Isotype control) / DA1E / Cell Signaling / Cat#3900S / https://www.cellsignal.com/datasheet.jsp?productId=3900&images=1&size=A4
Unconjugated / rat anti-mouse CD16/32 / BE0307 / BioXcell / Cat# BE0307 / https://d2a7cdyquyl45u.cloudfront.net/tds-sheets/BE0307-tds.pdf
Unconjugated / rat anti-mouse PD-L1 / 10F.9G2 / BioXcell / Cat# BE0101 / https://d2a7cdyquyl45u.cloudfront.net/tds-sheets/BE0101-tds.pdf
Unconjugated / rat anti-mouse CD8 / BE0061 / BioXcell / Cat# BE0061 / https://d2a7cdyquyl45u.cloudfront.net/tds-sheets/BE0061-

tds.pdf

Eukaryotic cell lines

Policy information about cell lines

Cell line source(s)	CT26 WT Cell line, ATCC (ATCC Cat# CRL-2638, RRID:CVCL_7256); MC-38 cell line Kerafast (Kerafast Cat# ENH204-FP RRID:CVCL_B288); B16-F10 cell line, ATCC (ATCC Cat# CRL-6475, RRID:CVCL_0159); 4T1 cell line, ATCC (ATCC Cat# CRL-2539, RRID:CVCL_0125).
Authentication	Cell lines obtained from commercial sources were cultured for a limited number of passages. The morphology and the growth pattern were strictly monitored as per manufacturers instructions.
Mycoplasma contamination	All cell lines used in this study were were tested for Mycoplasma contamination and are negative.
Commonly misidentified lines (See ICLAC register)	No commonly misidentified cell lines were used in the study

Animals and other organisms

Policy information about studies involving animals; ARRIVE guidelines recommended for reporting animal research

Laboratory animals	Animal experiments were performed with 8-12 week-old mice. C57BL/6J and BALB/c mice were purchased from the Jackson Laboratory. All mice were housed under a 12/12 light/dark cycle at 22-25°C and 30-70% humidity under specific pathogen-free conditions. Mice received sterilized rodent chow and water ad libitum. Gata6fl/fl mice were kindly provided by Dr. Medzhitov (Yale University) and bred in-house with Lyz2cre mice. Lyz2cre;Gata6fl/fl mice were subsequently bred with Gata6fl/fl to generate Cre+ (denoted as Mac-Gata6 KO) and Cre- (denoted as Mac-Gata6 WT) littermates. CCR2 KO (Ccr2Rfp/Rfp) mice were kindly provided by Richard M Ransohoff (Lerner Research Institute, Cleveland Clinic, Cleveland) and Israel F. Charo (University of California san Francisco, San Francisco). Gata6H2B-Venus reporter mice were kindly provided by Dr. Hadjantonakis (Memorial Sloan Kettering). Lyz2cre;Gata6fl/fl, Gata6fl/fl & Gata6H2B-Venus mice were back-crossed onto BALB/c background for at least 8 generations to achieve greater than 98% pure background. Single Nucleotide Polymorphisms (SNPs) testing was performed by Taconic Biosciences to confirm genetic purity. PD-L1 deficient mice were kindly provided by Dr. Arlene Sharpe (Harvard Medical School) as previously reported in Latchman, YE et al 2004, PNAS. Male and female mice were used for experiments. Mice were maintained in a specific pathogen-free facility at the University of Calgary Animal Resource Centre.
Wild animals	No wild animals were used in this study
Field-collected samples	No field-collected samples were included in this study
Ethics oversight	All experiments involving animals were approved by the University of Calgary Animal Care Committee and were in compliance with guidelines established by the Canadian Council for Animal Care.

Note that full information on the approval of the study protocol must also be provided in the manuscript.

Flow Cytometry

Plots

Confirm that:

- [x] The axis labels state the marker and fluorochrome used (e.g. CD4-FITC).
- [x] The axis scales are clearly visible. Include numbers along axes only for bottom left plot of group (a 'group' is an analysis of identical markers).
- [x] All plots are contour plots with outliers or pseudocolor plots.
- [x] A numerical value for number of cells or percentage (with statistics) is provided.

Methodology

Sample preparation	For isolating non-parenchymal cells from normal and metastases bearing livers, livers were perfused with HBSS, minced into small pieces digested in collagenase. Single-cell suspensions were generated by mechanical disruption through a 70-µm nylon mesh (BD Bioscience). Cellular debris and hepatocytes were removed by 33% isotonic Percoll (Sigma-Aldrich). Single-cell suspensions from peritoneal cavity were collected by lavaging the peritoneal cavities with cold HBSS. Dead cells were excluded using fixable viability dye (ebioscience). Cell surface-expressed molecules were stained after CD16/32 blocking. Intracellular and nuclear staining was performed by using the Foxp3 nuclear factor staining buffer set (ebioscience).
Instrument	The samples were run using a BD FACS Canto Cytometer (BD Biosciences)
Software	BD FacsDiva software was used for the acquisition of flowcytometry data and Flowjo software was used to analyze the data.
Cell population abundance	Cell sorting was not performed

Gating strategy	Gata6+ large peritoneal macrophages were pre-gated on singlet, live, CD45+, CD11b+ and then identified as F4/80hi and PKH26 or CD102+.

☒ Tick this box to confirm that a figure exemplifying the gating strategy is provided in the Supplementary Information.
